# Systematic Characterisation and Analysis of Lysyl Oxidase Family Members as Drivers of Tumour Progression and Multiple Drug Resistance

**DOI:** 10.1111/jcmm.70536

**Published:** 2025-04-03

**Authors:** Hongjin Liu, Xiaojiao Sun, Bingqi Dong, Jixin Zhang, Junling Zhang, Yanlun Gu, Lin Chen, Xiaocong Pang, Jingming Ye, Xin Wang, Zhuona Rong

**Affiliations:** ^1^ Department of Gastrointestinal Surgery Peking University First Hospital Beijing China; ^2^ School of Pharmaceutical Sciences, Peking University Beijing China; ^3^ Department of Pathology Peking University First Hospital Beijing China; ^4^ Department of Pharmacy Peking University First Hospital Beijing China; ^5^ Beijing Key Laboratory of Clinical Pharmacology and Translation of Innovative Drugs Peking University First Hospital Beijing China; ^6^ Department of Thyroid and Breast Surgery Peking University First Hospital Beijing China

**Keywords:** chemotherapy, drug response, endocrine therapy, immunotherapy, LOX, targeted therapy, tumour microenvironment

## Abstract

The intricacies of tumour microenvironment, particularly the extracellular matrix (ECM), underscore its pivotal function in modulating tumour progression and drug resistance. Among the key regulators of ECM remodelling and homeostasis, the lysyl oxidases (LOXs) emerge as promising therapeutic targets of tumour treatment. Despite their significance, a holistic evaluation of the LOX family's genomics and clinical implications across diverse cancer types remains elusive. Herein, this study aimed to investigate the correlation between LOX family expression and patient outcomes, drug responsiveness and tumour microenvironment (TME) characteristics in a cohort of 33 tumours based on The Cancer Genome Atlas (TCGA) database. Notably, patients exhibiting elevated LOX family expression suffer from worse prognosis and resistance to a spectrum of antitumor therapies, encompassing chemotherapy, endocrine therapy, targeted therapy and immunotherapy, in contrast to counterparts with subdued LOX family expression levels. Furthermore, enrichment analysis indicated that the LOX family fosters tumour progression and drug resistance. These findings were further validated by multiplex immunofluorescence staining in breast, gastric and rectal cancer, as well as breast cancer organoids. Altogether, this study unravels the intricate association between the LOX family and tumour progression, alongside multidrug resistance. We have gained further insights into the roles of LOX family genes in various tumour types, offering a novel avenue for future research into the relationship between LOX family genes and tumorigenesis.

## 1 Introduction

The tumour microenvironment (TME), which plays a pivotal role in cancer initiation and progression, comprises a diverse array of cellular components including fibroblasts, endothelial cells, immunocytes and adipocytes, as well as the extracellular matrix (ECM). Interaction between the ECM and tumour/stromal cells orchestrates fundamental processes in cancer development, spanning cell proliferation, migration, invasion, angiogenesis and immune evasion. These interactions serve multifaceted roles, encompassing structural support provision, local microenvironment modulation and acting as a reservoir for signalling molecules, growth factors and cytokines [1, 2]. Moreover, the ECM assumes a defensive role against cancer treatments and fosters tumour progression through macromolecular component alterations, enzymatic degradation and stiffness modulation [3]. ECM remodelling in numerous tumour tissues is typified by escalated collagen production and accumulation, alongside modifications in protein composition, functionality and cross‐linking. This remodelling process entails the activation of enzymes targeting specific ECM components as substrates, catalysing them to regulate tissue stiffness and cell‐matrix interactions via distinct biochemical and physical characteristics [4, 5]. Among these enzymes, the LOX family assumes a pivotal role in ECM remodelling.

The LOX family comprises five lysyl tyrosine quinone (LTQ)‐dependent copper amine oxidases in humans, consisting of LOX and lysyl oxidase‐like 1–4 (LOXL1‐4). These enzymes are characterised by a catalytic domain situated at conserved C‐terminal regions, enabling the oxidation of epsilon‐amino moieties of lysine on ECM components like elastin and collagens. Furthermore, the LOX family can be classified into two subfamilies based on their N‐terminal structure. Subfamily 1 includes LOX and LOXL1, while subfamily 2 comprises LOXL2, LOXL3 and LOXL4 [6, 7, 8, 9].

Multiple studies have conclusively evidenced the involvement of the LOX family in various tumour types [10, 11]. Consistently, the expression of LOX family members has been associated with poor prognoses in patients. Moreover, the LOX family significantly contributes to tumour progression through intracellular and extracellular mechanisms, including ECM remodelling, angiogenesis and immune cell infiltration. Simultaneously, numerous LOX inhibitors have been developed with the goal of serving as potential therapeutic agents for tumour treatment or fibrotic disease management. For example, PXS‐5505, a broad‐spectrum LOX inhibitor, has been reported to effectively suppress the functions of multiple lysyl oxidases and has exhibited safety in a phase 1 clinical trial [12, 13]. LOX and LOXL2 are genes upregulated by hypoxia, promoting tumour cell invasion and metastasis [14, 15].

Despite significant advances in cancer treatment, encompassing surgery, chemotherapy, radiotherapy, targeted therapy and immunotherapy, tumour recurrence and distant metastasis following systemic antitumor therapy remain primary drivers of cancer‐related mortality. A prior study underscored the role of the ECM in regulating tumour chemotherapy resistance, amplified by its induction of epithelial mesenchymal transition (EMT) and paclitaxel resistance in pancreatic cancer [16, 17], as well as its reduction of ovarian cancer cell sensitivity to platinum treatment [16]. Furthermore, ECM stiffness can influence cancer cell sensitivity to molecular targeted drugs, evidenced by the promotion of resistance in breast cancer cells to the HER2 inhibitor lapatinib and in melanoma cells to the BRAF inhibitor vemurafenib [18]. Moreover, the ECM may contribute to immune evasion through integrin‐dependent regulation of TGF‐β [19], yet its impact on immune surveillance and cancer immunotherapy is intricate and largely dependent on immune cell type. Although ECM‐remodelling enzymes, including those in the LOX family, have been definitively linked to promoting tumour progression, invasion and metastasis, further elucidation of the correlation and mechanism between the LOX family and tumour drug resistance is imperative for their potential utilisation as therapeutic options in antitumor treatment.

This study outlooks a comprehensive assessment of the genomics and clinical attributes of the LOX family across 33 solid tumours. The results unveiled a significant correlation between aberrant expression of the LOX family and the activation of cancer hallmark‐related pathways, alongside clinical survival outcomes. Moreover, this investigation delved into the association between LOX family member expression and the effectiveness of various antineoplastic treatments, including chemotherapy, targeted therapies and immunotherapy via multiplex immunofluorescence staining with pre‐treatment tumour specimens from breast, rectal and gastric cancer patients undergoing neoadjuvant therapy, as well as breast cancer organoids. These findings suggested that the upregulation of the LOX family not only correlated with worsened prognosis but may also foster the development of drug resistance in multiple tumours. Consequently, targeting the LOX family could emerge as a pivotal strategy for overcoming drug resistance.

## 2 Materials and Methods

### 2.1 Dataset

The UCSC Xena website (http://ualcan.path.uab.edu/analysis‐prot.html) was utilised to obtain RNA‐Seq data, gene mutation data and clinical data from the TCGA, GTEx and CCLE databases. Methylation data (HM450) and copy number alteration (CNA) data were retrieved from the cBioPortal database (http://www.cbioportal.org/). Immune cell infiltration analysis was conducted using data from the ImmuCellAI database (http://bioinfo.life.hust.edu.cn/ImmuCellAI#!/), TIMER2 database (http://timer.cistrome.org/) and previously published studies [20, 21].

### 2.2 Prognostic Analysis

K‐M and Cox univariate regression analyses were conducted to assess OS, DSS, PFI and DFI utilising the R packages ‘survminer’ and ‘survival’. The Kaplan–Meier curves were examined using both the median and optimal cut‐off values as thresholds.

### 2.3 The Tumour Microenvironment (TME), Immune Cell Infiltration, Tumour Mutation Burden (TMB) and Microsatellite Instability (MSI)

The relationships between the TME, immune cell infiltration and LOX family expression were assessed using the R packages ‘ggplot2’, ‘ggpubr’ and ‘ggExtra’. The correlation between the TMB, MSI and expression of LOX family members was determined through the Spearman method. The stromal score, immune score, ESTIMATE score and tumour purity were evaluated using the R package ‘ESTIMATE.’

### 2.4 Enrichment Analysis

Functional enrichment analyses, including Gene Ontology (GO), Kyoto Encyclopedia of Genes and Genomes (KEGG), gene set enrichment analysis (GSEA) and gene set variation analysis (GSVA), were conducted using the R packages ‘limma’, ‘org.Hs.eg.db’, ‘clusterProfiler’, ‘enrichplot’ and ‘GSVA’.

### 2.5 Drug Sensitivity Estimation

The CCLE, Genomics of Drug Sensitivity in Cancer (GDSC), Cancer Therapeutics Response Portal (CTRP) and PRISM Repurposing datasets were utilised to compile expression data and drug sensitivity information for cancer cell lines (CCLs). The R packages ‘oncoPredict’ and ‘pRRophetic’ were used to calculate the IC_50_ and AUC values for each drug.

### 2.6 Immunotherapeutic Analyses

The GSE35640, GSE61676, GSE78220, GSE135222 and GSE176307 datasets were retrieved from the Gene Expression Omnibus (GEO) database (https://www.ebi.ac.uk/ega/), while information on IMvigor210 was accessed through the R package ‘IMvigor210CoreBiologies’. Other relevant data sources included published literature [22, 23, 24].

### 2.7 Multiplex Immunohistochemistry (mIHC)

Pathological and clinical data were collected from patients who were diagnosed with triple‐negative breast (*n* = 20), gastric (*n* = 2) or rectal (*n* = 2) cancer and who underwent standard preoperative therapy, including chemotherapy, immunotherapy and targeted therapy, at Peking University First Hospital. All samples were procured prior to the initiation of treatment. Subsequently, based on postoperative Miller‐Payne pathological grading, a cohort of 20 triple‐negative breast cancer patients who underwent standard neoadjuvant chemotherapy were analysed, with 10 patients exhibiting pathological complete response (CR) (M‐P Grade 5) and the remaining 10 patients showing pathological poor response (PR) (M‐P Grade 1). Patients diagnosed with gastric and rectal cancer were screened based on CAP pathological grading. Two individuals with gastric cancer underwent standard conversion chemotherapy and immunotherapy, resulting in one achieving pathological CR (CAP Grade 0) and the other showing pathological PR (CAP Grade 3). Additionally, two patients with gastric cancer received standard conversion chemotherapy along with targeted therapy, one demonstrating pathological CR (CAP Grade 0) and the other showing pathological PR (CAP Grade 3). The fulvestrant‐resistant (n‐2) and sensitive (*n* = 2) breast cancer organoids, along with their corresponding clinical data, were obtained from Beijing K2 Oncology Technology Co. Ltd., and the CytoMap multidimensional database platform. Drug sensitivity analysis was conducted on all organoids. Specific details regarding the medication and clinical data for each patient and organoid can be found in Table S1. This study was conducted in accordance with the principles outlined in the Declaration of Helsinki and was approved by the Ethics Committee on Human Research of Peking University First Hospital under authorization number 2018–15.

The primary antibodies used for immunohistochemical staining were against LOX, LOXL2, α‐SMA, CD68 and CD206. The sections were visualised by staining with appropriate secondary antibodies followed by incubation with an avidin‐biotin‐peroxidase complex. Organoid pathological sections were incubated with antibodies against LOX (ab174316, Abcam) and LOXL2 (ab314140, Abcam). Multiplex fluorescent immunohistochemical staining of the sections was performed using antibodies targeting α‐SMA (GB12045, Servicebio), CD68 (GB113150, Servicebio) and CD206 (GB115273, Servicebio). The tumour areas were marked by a pathologist. These selected regions are referred to as regions of interest (ROIs). Immunofluorescence was quantified by the fluorescence area fraction and mean fluorescence intensity.

### 2.8 Statistical Analyses

The data are presented as the mean ± standard error of the mean (SEM). *Student's t test* or analysis of variance (ANOVA) was employed to assess differences between groups, while Pearson correlation analysis was utilised to examine relationships. Statistical tests were carried out using GraphPad Prism software version 8.0.2 (GraphPad Software, San Diego, CA, USA). Statistical significance was defined as *p* < 0.05, with * indicating *p* < 0.05, ** indicating *p* < 0.01, *** indicating *p* < 0.001 and **** indicating *p* < 0.0001, while ns denotes nonsignificant results.

## 3 Results

### 3.1 Expression Analysis of LOX Family Members in pan‐Cancer

**FIGURE 1:**
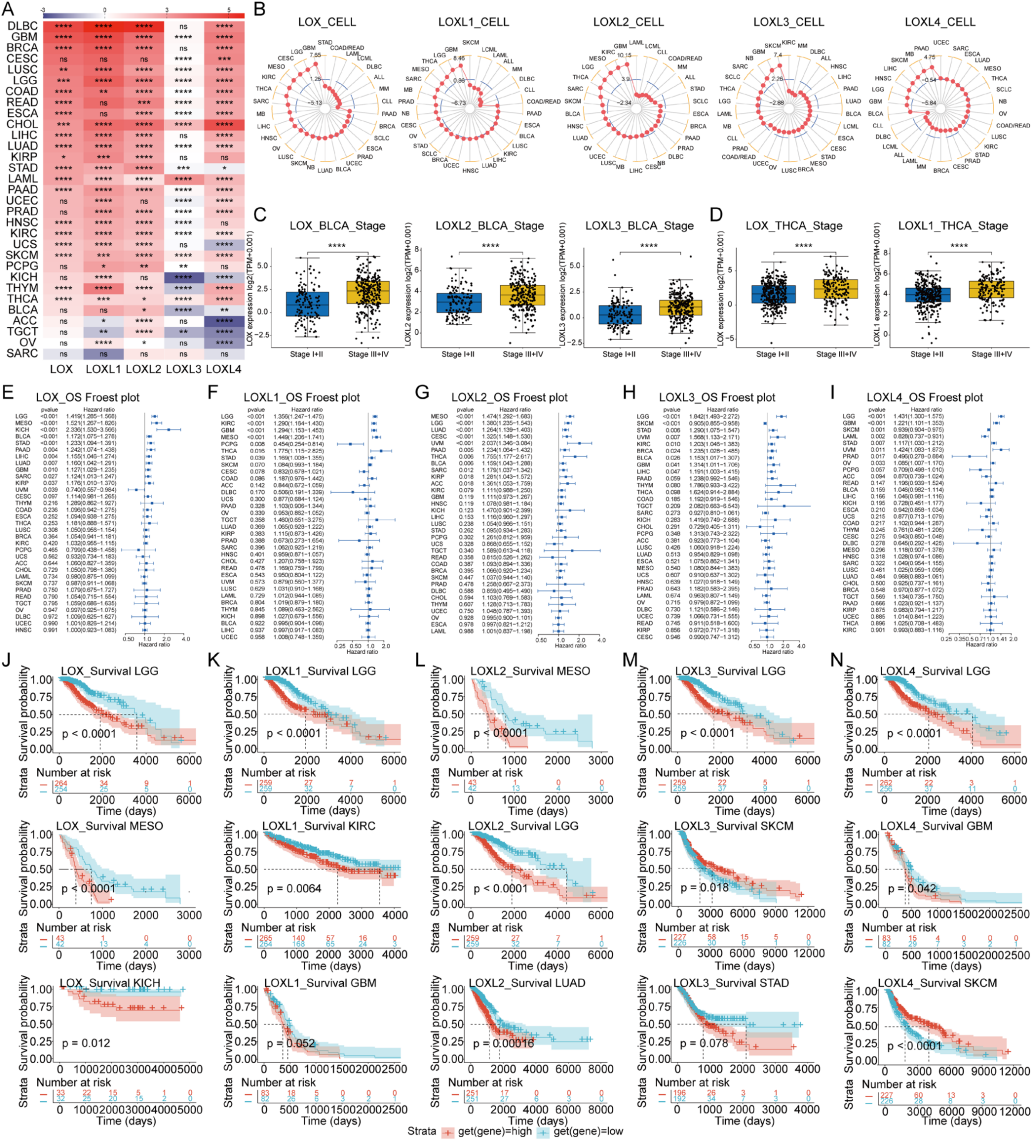
LOX family expression and its correlation with prognosis. (A) Comparison of LOX family expression in tumour tissues from The Cancer Genome Atlas (TCGA) and normal tissues from the Genotype‐Tissue Expression (GTEx) database. (B) The expression of the LOX family in tumour tissues from the Cancer Cell Line Encyclopedia (CCLE). (C, D) The expression of LOX, LOXL2 and LOXL3 in bladder urothelial carcinoma (BLCA) and LOX and LOXL1 in thyroid carcinoma (THCA) at different clinical stages from TCGA. (E–I) Association of LOX family expression with overall survival (OS) according to univariate Cox analysis. (J–N) Association of LOX family expression with overall survival (OS) according to Kaplan–Meier analysis. *p < 0.05; **p < 0.01; ***p < 0.001 and ****p < 0.0001; ns, nonsignificant.

First, we conducted an investigation into the expression levels of LOX family involved in 33 tumour types from The Cancer Genome Atlas (TCGA), 31 normal tissues from the Genotype‐Tissue Expression (GTEx), and human cell lines representing 30 cancer types from Cancer Cell Line Encyclopedia (CCLE). The results of TCGA indicated that LOX, LOXL1, LOXL2, LOXL3 and LOXL4 expressed highest in KIRC, SARC, SARC, PCPG and CHOL, as well as lowest in LAML, LAML, LAML, KICH and LGG, respectively. For normal tissues from GTEx, LOX, LOXL1, LOXL2, LOXL3 and LOXL4 were highest in adipose tissue, blood vessel, adipose tissue, spleen and salivary gland, as well as lowest in blood, bone marrow, blood, liver and blood (Figure S1). It was found that the expression of the LOX family was notably regulated in 30 tumour tissues when compared with normal tissues, including PAAD, GBM, LAML, DLBC, THYM, LIHC, CHOL, STAD, HNSC, UCS, KIRC, ESCA, COAD, READ, CESC, OV, ACC, PRAD, UCEC, BLCA, BRCA, LUSC, PCPG, LGG, TGCT, KICH, LUAD, KIRP, THCA and SKCM (please see Table S2 for the abbreviations and full names of the 33 cancers in TCGA), whereas there was no significant regulation in SARC (Figure 1A and Table S3). LOX was higher than that of the corresponding normal tissue in majority of tumours, as these 15 kinds of tumours: DLBC, KIRC, THYM, PAAD, CHOL, GBM, STAD, ESCA, LAML, LIHC, HNSC, UCS, COAD and READ. In contrast, there were still 7 tumours: LUSC, BRCA, LGG, LUAD, KIRP, THCA and SKCM, expressed lower in tumour. Regrettably, a combinatory analysis of MESO and UVM was not available due to the lack of normal organisation data for them in GTEx. In addition, we evaluated the LOX family expression in tumours and their paired adjacent tissues in TCGA, which showed that the produced results were consistent with the combined analysis of TCGA and GTEx (Table S3). Concerning cancer cell lines, the expression of LOX, LOXL1, LOXL2, LOXL3 and LOXL4 was highest in GBM, GBM, GBM, SKCM and PAAD, while lowest in CLL, CLL, LCML, DLBC and CLL, respectively (Figure 1B).

To determine the expression of LOX family accurately, we further assessed the clinical significance of LOX family expression at different TNM stages, manifesting that LOX expression was higher in advanced tumour types:BLCA, THCA, KIRC, LUAD, LIHC and so on. Unsurprisingly, significant stage‐specific differences in the expression of LOX, LOXL2 and LOXL3 were observed in BLCA (Figure 1C), with LOX and LOXL1showing higher expression in the advanced stages of THCA (Figure 1D). Additional expression level details at TNM stages can be found in Table S4.

### 3.2 Prognosis Analysis of LOX Family in Cancer Patients

LOX family was highly regulated in a variety of tumours. In order to explore whether the expression level of LOX family correlated with patient survival, univariate Cox and Kaplan–Meier analyses were employed to assess the prognostic significance of the LOX family in pan‐cancer patients. As the results were shown that high expression of the LOX family emerged as a significant risk factor for overall survival (OS), disease‐specific survival (DSS) and progression‐free interval (PFI) in most tumours. Notably, elevated LOXL2 expression consistently correlated significantly with poor prognosis across all tumour types. Conversely, certain members of the LOX family exhibited protective effects in specific tumours, such as LOX in OS for UVM, LOXL1 in OS, DSS and PFI for PCPG, LOXL3 and LOXL4 in OS, DSS and PFI for SKCM, LOXL3 in PFI for OV, LOXL4 in OS, DSS and PFI for PRAD, LOXL4 in OS for LAML, and LOXL4 in PFI for THYM and UCS (Figure 1E–I). Additionally, concerning disease‐free interval (DFI), both LOX and LOXL2 were identified as risk factors, whereas LOXL1 was found to be a protective factor (Figure S2 and Table S5).

The Kaplan–Meier curves utilised both the median and optimal cut‐off values as thresholds for statistical analysis. The results revealed that employing the optimal cut‐off value for grouping resulted in significantly greater differences in tumour types between the high‐expression and low‐expression groups compared to using the median as the cut‐off point. Generally, tumours with high expression of the LOX family exhibited a poorer prognosis in terms of OS, DFI, DSS and PFI. Intriguingly, LOXL4 exhibited a protective effect on half of the tumours in terms of the PFI. (Figure 1J–N and Table S6).

### 3.3 The Expression of LOX Family Correlated With Copy Number Alterations (CNAs) and Hypomethylation

**FIGURE 2:**
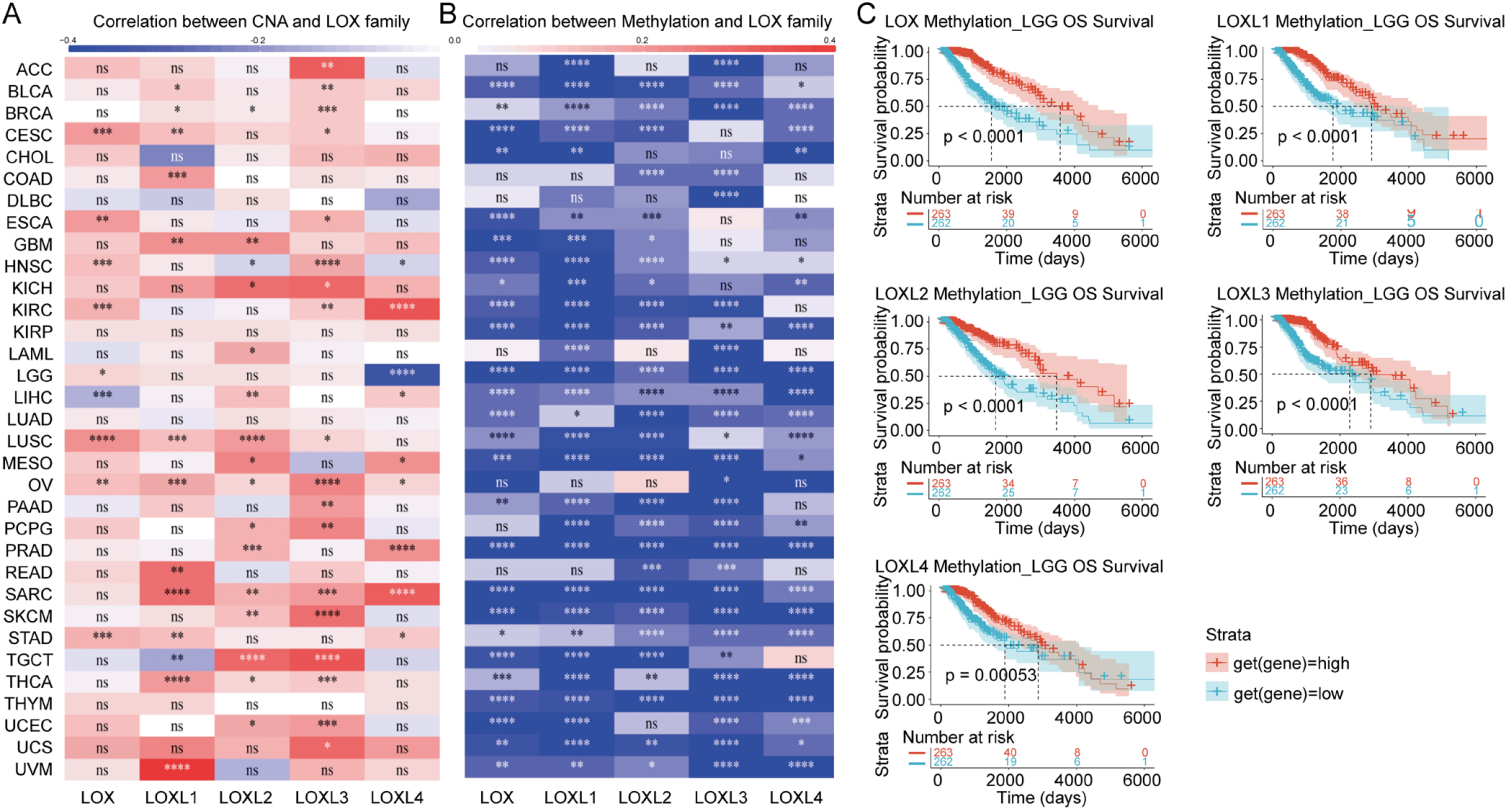
Relationship between the LOX family and copy number alterations (CNAs) or methylation, as well as the correlation between methylation and prognosis. (A) The correlation between the LOX family and CNA. (B) The correlation between the LOX family and methylation. (C) The correlation between methylation and prognosis. *p < 0.05; **p < 0.01; ***p < 0.001 and ****p < 0.0001; ns, nonsignificant.

The goal is to characterise functional consequences of DNA copy number and methylation alterations in tumours and to facilitate screening for drivers contributing to the expression of LOX family. The results revealed a predominantly positive correlation between CNA and LOX family members in most tumours, with only a few exceptions noted, such as LOX in LIHC, LOXL1 in TGCT and LOXL4 in HNSC and LGG (Figure 2A). Conversely, a negative association was evident between methylation and the LOX family across all tumours, with statistically significant differences (Figure 2B). Furthermore, Kaplan–Meier curves were generated to assess the prognostic implications of methylation levels, indicating a favourable prognosis in LGG (Figure 2C) and other tumours (Table S7) associated with high methylation of LOX family members.

### 3.4 LOX Family Expression Correlated With EMT, Immune Response and Cellular Metabolism Pathways

**FIGURE 3:**
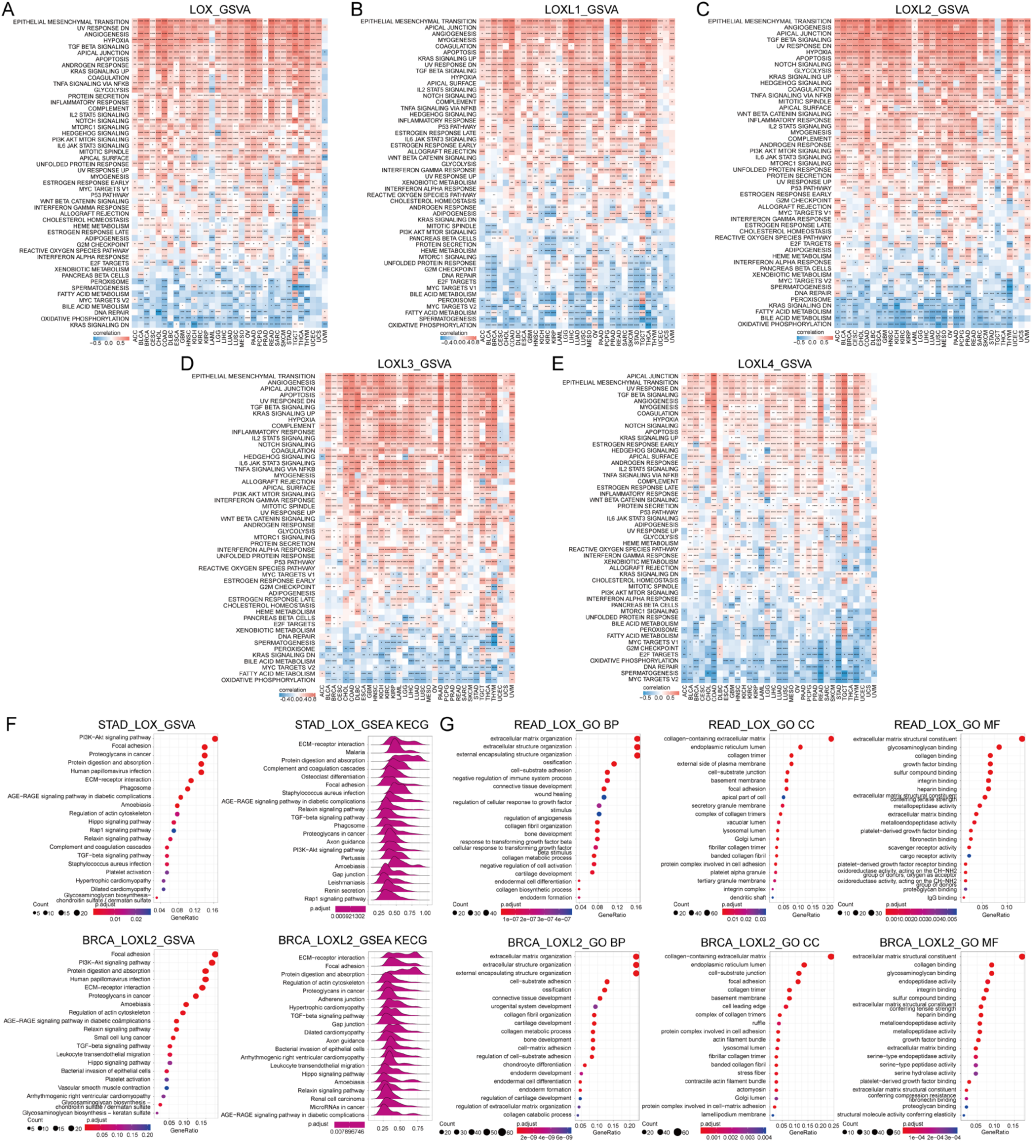
The enrichment analysis of the LOX family. (A–E) GSVA of the LOX family. (F, G) GO, KEGG and GSEA analyses of the LOX family. BP, biological process; CC, cellular component; GO, gene ontology; GSEA, gene set enrichment analysis; GSVA, gene set variation analysis; KEGG, Kyoto Encyclopedia of Genes and Genomes; MF, molecular function. *p < 0.05; **p < 0.01; ***p < 0.001 and ****p < 0.0001.

Gene Set Variation Analysis (GSVA) was employed to investigate potential pathways involving the LOX family. Our findings suggested a strong positive association between the expression levels of LOX family members and pathways related to EMT, including TGF‐, Notch, WNT/‐catenin, Hedgehog and TNF‐/NF‐ signalling. Moreover, these pathways were enriched in chemotherapy resistance, PI3K/AKT/mTOR, KRAS, Notch and TNF‐/NF‐ signalling. Furthermore, several immune‐related pathways, such as the P53 pathway, glycolysis, apoptosis, inflammatory response, IL2/STAT5, hypoxia and IL6/JAK/STAT3 signalling pathways, displayed positive correlations with LOX family expression, suggesting a potential increase in immune cell infiltration in patients with elevated LOX family expression. Conversely, pathways related to cellular metabolism (peroxisome, oxidative phosphorylation, bile acid metabolism and fatty acid metabolism), MYC target V2 and DNA repair exhibited negative correlations with LOX family expression (Figure 3A–E). Additionally, the Gene Ontology (GO), Kyoto Encyclopedia of Genes and Genomes (KEGG) and Gene Set Enrichment Analysis (GSEA) results provided supplementary evidence supporting the robust relationships between LOX family expression and EMT, immune response and cellular metabolism (Figure 3F,G).

### 3.5 LOX Family Modulated Tumour Microenvironment Across Cancer Types

**FIGURE 4:**
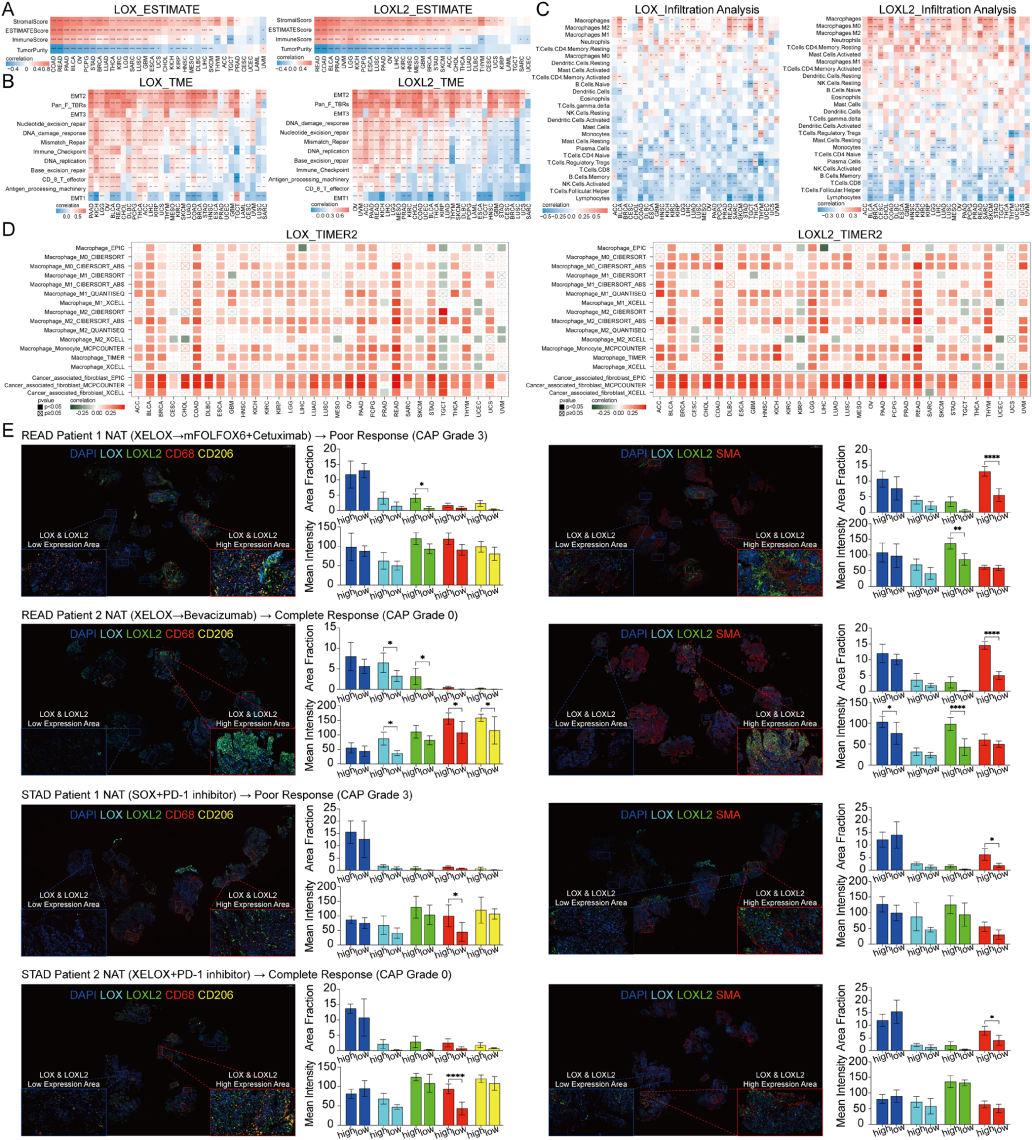
Relationship between the LOX family and the tumour microenvironment (TME) and immune infiltration analysis, as well as multiplex immunohistochemistry (mIHC), of gastric and rectal cancer. (A) Correlations between the LOX family and immune score, stromal score, estimate score and tumour purity. (B) The correlation between LOX expression and TME‐related signatures or pathways. (C) The correlation between LOX expression and immune infiltration. (D) The correlation between LOX expression and immune infiltration according to the TIMER2 database. (E) mIHC results showing the fluorescence area fraction, mean fluorescence intensity and immunofluorescence colocalization in gastric and rectal cancer tissues. *p < 0.05; **p < 0.01; ***p < 0.001 and ****p < 0.0001; ns, nonsignificant.

Various types of tumours exhibit distinctive tumour microenvironments (TMEs) composing tumour cells, immune cells and stromal cells. In order to explore the impact of the LOX family on the TME, we conducted an evaluation of TME scores. Our findings unveiled a positive correlation between the expression of LOX family members and stromal score, ESTIMATE score and immune score in the majority of tumours. However, we also identified a negative correlation with tumour purity, indicating a compromised immune response. Intriguingly, LOX in the UVM group, LOXL1 and LOXL4 in the LAML group and LOXL4 in the THYM group displayed contradictory results (Figure 4A and Figure S3). The results showed the potential involvement of the LOX family in tumour progression through its modulation of TME across diverse cancer types. Furthermore, an investigation into the interplay between the LOX family and TME‐associated signatures or pathways revealed a consistent positive correlation across all signatures, with the exception of a negative correlation with EMT1. Specifically, heightened expression of the LOX family in the majority of tumours was linked to increased levels of EMT2, Pan_F_TBRs (panfibroblast TGF‐response signature), EMT3 and other factors (Figure 4B and Figure S3).

### 3.6 LOX Family and Immune Microenvironment: Multi‐Database Analysis and mIHC Validation

The TME score indicated a strong association between the LOX family and immune score. Subsequently, we conducted an analysis utilising three distinct databases–TIMER2, ImmuCellAI and published studies to investigate the correlation between the LOX family and components of the immune microenvironment, including immune cells, factors and genes. The findings from these databases consistently demonstrated a notable relationship between the LOX family and a substantial proportion of immune cells across various tumour types. Specifically, in the TIMER2 database, the LOX family exhibited a positive correlation with numerous immune cells present in tumours, particularly cancer‐associated fibroblasts (CAFs) and tumour‐associated macrophages (TAMs) (Figure 4D). Similarly, in the ImmuneCellAI database, macrophages, iTregs and monocytes displayed a positive correlation with the LOX family in pan‐cancer analysis, although certain immune cells, such as B cells and CD8^+^ T cells, exhibited negative correlations. These findings are congruent with and supported by published studies, thereby reinforcing the validity and reliability of the aforementioned databases (Figure 4C and Figure S3).

mIHC was performed on tumour specimens obtained from patients diagnosed with gastric and rectal cancer, aiming to investigate the association between the expression levels of LOX and LOXL2 and the presence of CAFs expressing α‐SMA, as well as TAMs expressing CD68 and CD206. The results indicated a positive correlation between elevated levels of LOX and LOXL2 expression and increased levels of α‐SMA, CD,68 and CD206 in tumour regions, although some distinction did not attain statistically significant. Furthermore, the fluorescence area fraction and intensity notably increased in regions displaying high LOX and LOXL2 expression compared to those with low expression level of these proteins. CAFs and TAMs were found to be enriched in tumour regions characterised by enhanced expression levels of LOX and LOXL2 compared to regions with lower expression levels of these proteins (Figure 4E). Additionally, immunofluorescence colocalization experiments demonstrated that LOX and LOXL2 did not co‐localised with α‐SMA, CD68 and CD206 (Figure S4).

### 3.7 LOX Family Contributed to Chemotherapy/Endocrine/Targeted Therapy Resistance

**FIGURE 5:**
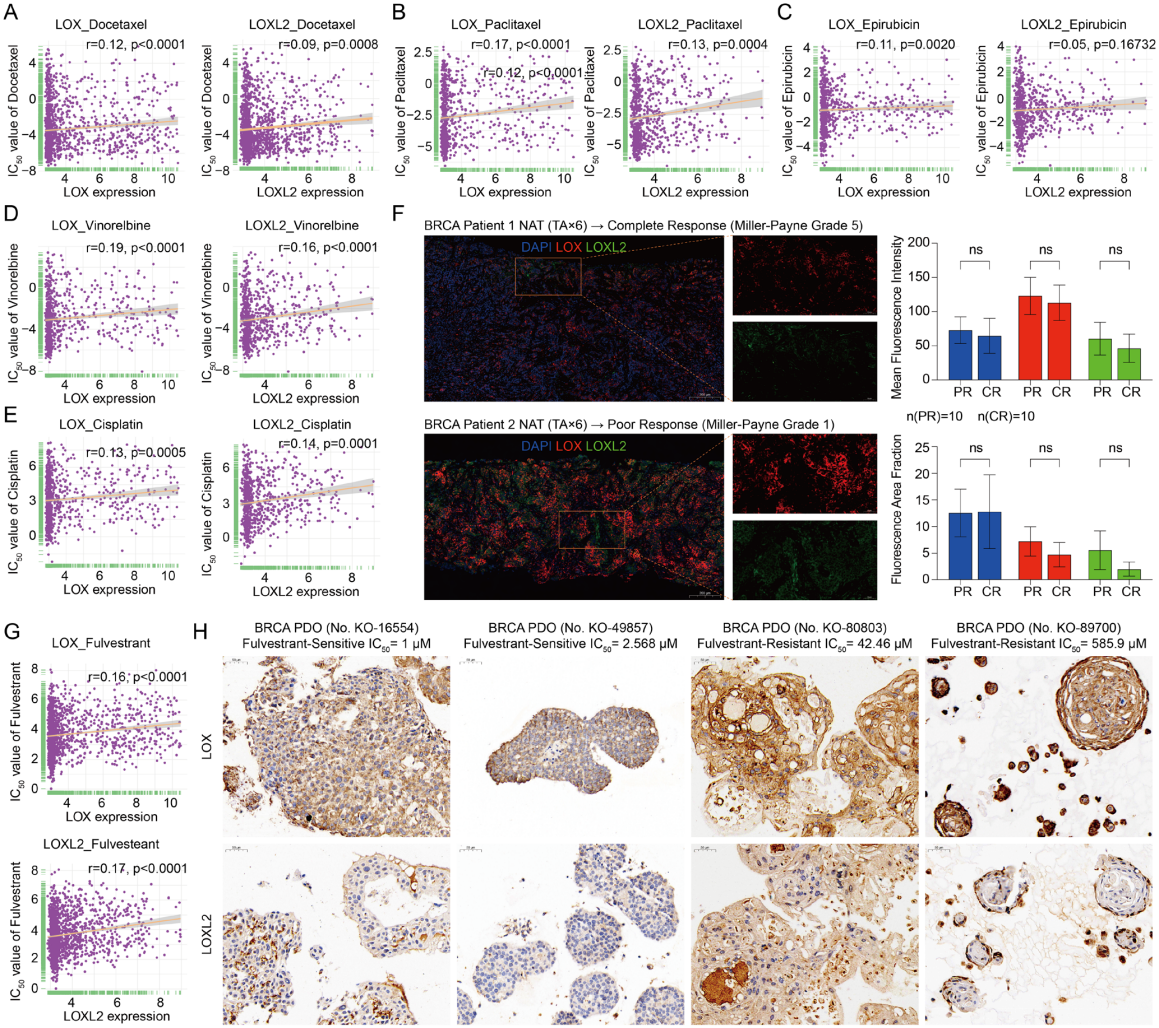
Relationships between LOX/LOXL2 expression and chemotherapy/endocrine therapy response, as well as between multiplex immunohistochemistry (mIHC) and immunohistochemistry (IHC), in breast cancer patients. (A‐E) Relationships between LOX/LOXL2 and the IC_50_ values of docetaxel, paclitaxel, epirubicin, vinorelbine and cisplatin. (F) mIHC results showing the fluorescence area fraction and mean fluorescence intensity in triple‐negative breast cancer patients. (G) The relationship between LOX/LOXL2 and the IC_50_ values of fulvestrant. (H) The IHC results in breast cancer organoids. CR, complete response; ns, nonsignificant; PR, poor response.

Further investigation was conducted to explore the interplay between the LOX family and the IC_50_ values of 192 anti‐tumour drugs. The findings indicated that elevated levels of LOX, LOXL1, LOXL2 and LOXL4 in patients might contribute to chemotherapy resistance, encompassing commonly prescribed drugs such as docetaxel, paclitaxel, epirubicin, vinorelbine, cisplatin, cyclophosphamide and oxaliplatin (Figure 5A–E and Table S8). mIHC was carried out on a cohort of 20 patients diagnosed with triple‐negative breast cancer who underwent neoadjuvant chemotherapy. The findings indicated a reduction in the fluorescence area fraction and mean fluorescence intensity of LOX and LOXL2 in the tumour region of individuals showing sensitivity to neoadjuvant chemotherapy compared to those exhibiting resistance, although statistical significance was not achieved (Figure 5F). Nonetheless, a noticeable trend emerged, suggesting an inverse relationship between the expression levels of LOX and LOXL2 and the efficacy of chemotherapy. Concurrently, endocrine therapy assumed a pivotal role in the comprehensive treatment of breast cancer. Our findings revealed a negative correlation between the expression of LOX family genes, particularly LOX, LOXL1, LOXL2 and LOXL4 and the efficacy of commonly employed endocrine drugs, such as tamoxifen, fulvestrant and CDk4/6 inhibitors (palbociclib and ribociclib) (Figure 5G and Table S8). Immunohistochemistry (IHC) depicted elevated levels of LOXL2 in fulvestrant‐resistant breast cancer organoids compared to fulvestrant‐sensitive organoids, with no significant variance in LOX expression (Figure 5H).

The realm of molecular targeted therapy within clinical practice was undergoing swift advancements, where initial drug responses often showcased sensitivity but eventually developed into drug resistance. This study aimed to evaluate the relevance of the LOX family concerning molecular targeted drugs. Examination of the IC_50_ of 192 drugs unveiled a positive correlation between the expression of LOX, LOXL1, LOXL2 and LOXL4 and the IC_50_ values of most molecular targeted drugs. These included AKT inhibitors (afuresertib, ipatasertib and urosertib), Bcl‐2 inhibitors (navitoclax and venetoclax), BRAF inhibitors (dabrafenib), CDK4/6 inhibitors (palbociclib), HDAC inhibitors (entinostat and vorinostat), PARP inhibitors (niraparib, olaparib and talazoparib) and PI3K inhibitors (alpelisib, buparlisib and taselisib). Moreover, our findings indicated that patients with elevated expression levels of LOX, LOXL1, LOXL2 and LOXL3 might manifest resistance to EGFR inhibitors such as afatinib, erlotinib and gefitinib. Conversely, individuals with high levels of LOXL3 might exhibit sensitivity to certain molecular targeted drugs, including Bcl‐2 and HDAC inhibitors (Table S8).

### 3.8 LOX Family Correlated With Immunotherapy Response

Tumour mutational burden (TMB) and microsatellite instability (MSI) status have emerged as pivotal biomarkers for gauging the response to immune checkpoint inhibitors (ICIs). Initially employed to probe the relationship between the LOX family and immunotherapy response, TMB and MSI exhibited significant correlations with the LOX family in a specific subset of tumours, predominantly showcasing a negative association (Figure S3 and Table S9). Specifically, in COAD, LOX, LOXL1 and LOXL3 displayed positive correlations with TMB, whereas in SKCM and SARC, LOX, LOXL1 and LOXL2 exhibited negative associations with MSI and positive associations with SARC. This suggested a hypothesis that in certain cancer types, patients with heightened expression of LOX family members might exhibit resistance to immunotherapy.

**FIGURE 6:**
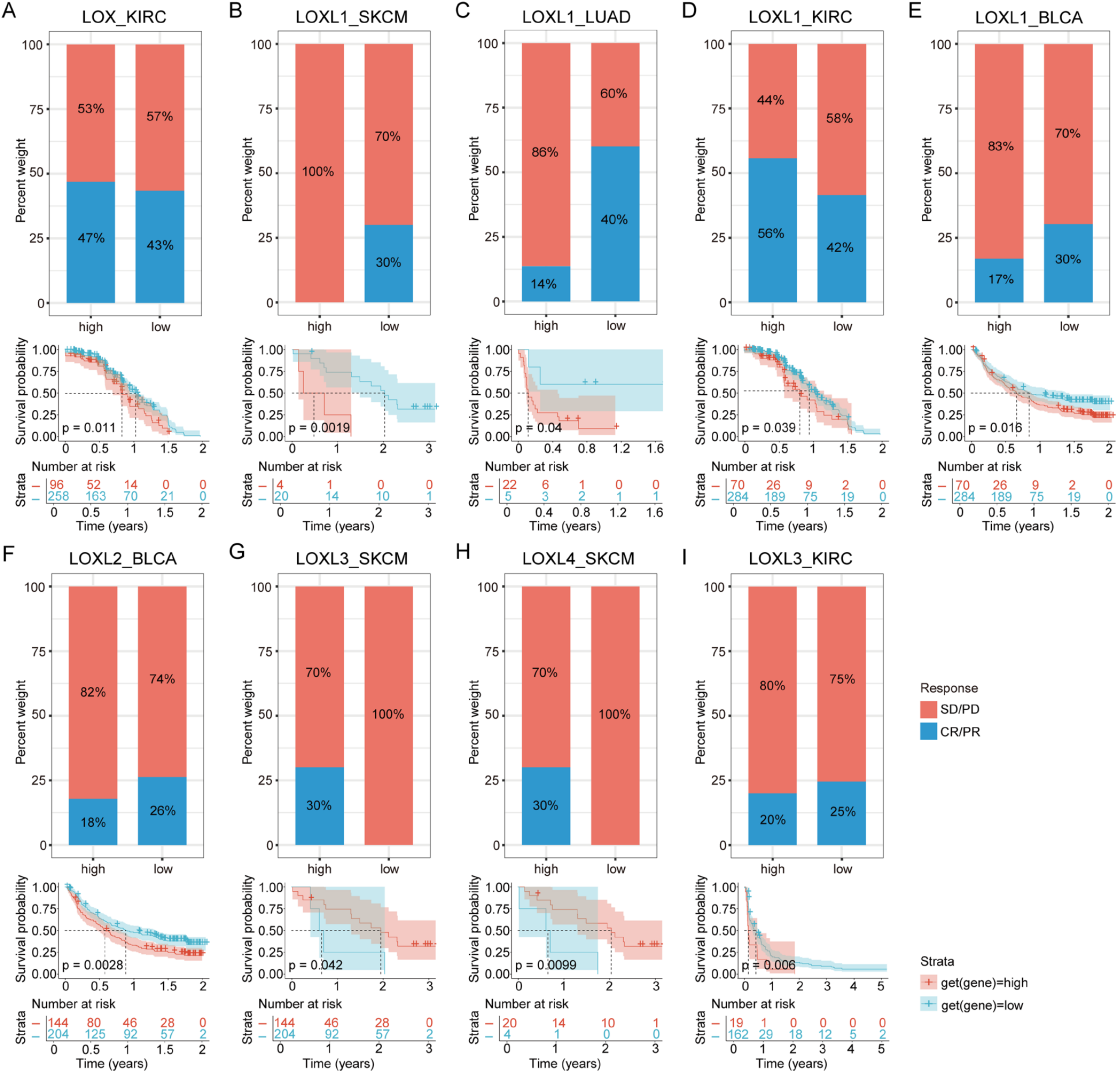
The relationship between the LOX family and immunotherapy response. (A) Relationships between LOX and PD‐1/PD‐L1 in KIRC. (B–E) Relationships between LOXL1 and PD‐1/PD‐L1 in SKCM, LUAD, KIRC and BLCA. (F) Relationships between LOXL2 and PD‐1/PD‐L1 in BLCA. (G‐H) Relationships between LOXL3/LOXL4 and PD‐1/PD‐L1 in SKCM. (I) Relationships between LOXL3 and PD‐1/PD‐L1 in KIRC.

To validate whether patients with elevated LOX family expression in specific cancer types were indeed resistant to immunotherapy, we screened independent datasets pertaining to programmed death 1 (PD‐1)/programmed death ligand 1 (PD‐L1) datasets from SKCM (GSE78220 and Riaz2017), LUAD (GSE135222), BLCA (IMvigor210CoreBiologies) and KIRC (NCT02684006 and PMID32472114). The high expression of LOX, LOXL1 and LOXL3 in KIRC was significantly correlated with shortened survival, and elevated expression of LOX and LOXL1 correlated with increased objective response rates (ORRs), while high LOXL3 expression showed a negative association with ORRs (Figure 6A,D,I). Similarly, elevated LOXL1 in SKCM, LUAD and BLCA and elevated LOXL2 in BLCA were associated with reduced ORRs and poorer survival (Figure 6B,C,E,F). Conversely, SKCM patients with elevated expression levels of LOXL3 and LOXL4 exhibited higher ORRs and significantly prolonged survival compared to cases with lower expression levels (Figure 6G,H).

## 4 Discussion

The LOX family has been established to play intricate and paradoxical roles in various cancers, encompassing tumour suppression, proliferation, migration, invasion and metastasis. Through comprehensive analysis of multiple omics data and rigorous validation of clinical data, our findings not only underscored the robust association between the upregulation of LOX family expression and unfavourable prognosis, as well as poor response to chemotherapy, targeted therapy and immunotherapy but also revealed numerous potential mechanisms governing LOX family regulation within the cancer milieu.

Significant correlations between the expression of the LOX family and tumour progression have been identified. Our study unveiled an increased expression of LOX and LOXL1‐3 across various cancer types, whereas LOXL4 expression demonstrated an inverse trend. Moreover, elevated LOX family expression served as an indicator of poor prognosis (OS, DFI, DSS and PFI) in tumour patients, such as LGG, MESO, LUAD, STAD, PAAD, BRCA, COAD, READ and LIHC. Notably, the expression of LOX and LOXL2 was most closely associated with poor prognosis. These findings echoed similar conclusions reported in prior studies, indicating that high expression of the LOX family, particularly LOX and LOXL2, correlated with adverse survival outcomes in breast cancer (especially in ER‐negative or triple‐negative patients) [25, 26, 27, 28, 29, 30], gastric cancer [31, 32, 33, 34], lung cancer [35, 36], colorectal cancer [37, 38], glioma [39] and pancreatic cancer [40, 41].

The LOX family plays key roles in tumorigenesis, metastasis and therapeutic resistance, and their biological effects are mainly mediated by a complex network of signalling pathways and transcription factor regulation. GSVA results in our study showed that classical EMT pathways such as TGF‐β, Notch and WNT/β‐catenin were significantly enriched in LOX family high‐expressing tumours, which was highly consistent with previous finding that the TGF‐β/LOX/Snail axis drives EMT [42]. Notably, these pathways were significantly co‐activated with chemoresistance at the same time, suggesting that LOX may promote treatment resistance through a dual mechanism: first, by enhancing tumour cell plasticity through EMT, and second, by maintaining survival signalling through PI3K/AKT/mTOR pathway. This finding provides a mechanistic explanation for the reduced sensitivity of LOX‐high tumours to platinum/paclitaxel drugs in the clinic and supports the strategy of combining LOX inhibitors with AKT inhibitors to overcome drug resistance. Although pro‐inflammatory signals such as IL6/JAK/STAT3 and IL2/STAT5 were positively correlated with LOX expression, previous studies showed that the increased immune infiltration in LOX‐high tumours was dominated by M2‐type macrophages (TAMs) and regulatory T cells (Tregs) rather than cytotoxic T cells [43]. This phenotypic paradox may be due to LOX‐induced upregulation of PD‐L1 via the HIF‐1α/NF‐κB axis [44] and a shift in the Th1/Th2 immune balance towards Th2 due to persistent STAT3 activation. LOX enhances the hypoxia adaptation of tumour cells by upregulating HIF‐1α, which in turn activates the NF‐κB pathway and drives the secretion of inflammatory factors (e.g., IL‐6, TNF‐α), further recruiting immunosuppressive cells (e.g., TAMs, MDSCs) to form a pro‐cancer TME [44, 45]. Thus, the LOX‐high microenvironment, although signatured by ‘immune activation’, is actually an immune‐evasion state, which explains why such patients may benefit from PD‐1 inhibitors in combination with LOX‐targeted therapy.

The TME and the heterogeneity of tumour cells were associated with the emergence of drug resistance. According to our research, the LOX family was involved in both the cellular and noncellular elements of the TME. As an important noncellular component of the TME, the ECM served as a physical barrier to drug transport and dissolution [46]. We found that the LOX family primarily modified integrin focal adhesion, growth factor receptor signalling, survival‐related pathways, and the covalent cross‐linking of collagen and elastin. These modifications impacted drug resistance through ECM deposition and tissue stiffness. Previous studies have confirmed that fibrocollagen expression in ovarian cancer cells might be associated with resistance to paclitaxel and topotecan [47], and that high expression of laminin 332 also indicated resistance to doxorubicin and sorafenib [48]. Additionally, LOX inhibition synergized with gemcitabine to kill tumour cells by altering stroma, which in the case of pancreatic cancer resulted in reduced fibrillar collagen and increased vasculature [49]. Hypoxia is another significant noncellular component of TME that affected medication effectiveness. Direct transcriptional targets of HIF‐1, LOX and LOXL2, were activated by hypoxia and were involved in the molecular process of E‐cadherin suppression, a characteristic feature of EMT [15]. This result was highly consistent with our findings that hypoxia was positively linked with LOX family expression in all types of cancer.

As one of the main components of TME, CAFs provide functional assistance and drug resistance by secreting ECM proteins [50, 51]. Prior research demonstrated that LOXL2 released by tumours induced ECM remodelling, leading to heightened stromal stiffness, and stimulated surrounding CAFs via integrin‐mediated focal adhesion kinase (FAK) activation [52] or extracellular signal‐regulated kinase (ERK) activation [53]. Our results showed that in gastric and rectal cancer specimens, tumour areas with high expression of LOX and LOXL2 also exhibited significant expression of α‐SMA, a specific surface marker for CAFs. Notably, there was a symbiotic link between CAFs and TAMs. Through the SDF‐1/CXCR4 axis, CAFs contributed to the recruitment and differentiation of monocytes in an immunosuppressive phenotype in a prostate cancer model. Conversely, SDF‐1‐polarised TAMs supported the development and proliferation of CAFs, which aided in immunological evasion. M2 macrophages and CAFs interact in both directions; the former could cause fibroblasts to undergo a mesenchymal‐mesenchymal transition (MMT), which increased the responsiveness of the latter [54]. According to our research, regions of tumours with high LOX and LOXL2 levels also had higher expression of CD68 and CD206, which were known to be markers of macrophages and M2 macrophages, respectively. Through enrichment and infiltration analyses, we observed a strong positive correlation between the expression of the LOX family and that of CAFs and TAMs. The complex interaction among cancer cells, TAMs and CAFs increased the motility of tumour cells, which in turn encouraged drug resistance and facilitated the spread of metastatic lesions.

Resistance to several chemotherapies and targeted treatments is strongly correlated with high LOX family expression, as demonstrated by the measurement of IC_50_ values for antitumour medicines. Additionally, our findings revealed that patients with triple‐negative breast cancer receiving neoadjuvant chemotherapy showed lower levels of LOX and LOXL2 expression in their tumour tissues than those who were sensitive to taxanes, anthracyclines and platinum agents. ICI treatment had advanced significantly as a result of immunotherapies targeting the PD‐1/PD‐L1 axis. Results from many separate patient cohorts treated with PD‐1/PD‐L1 inhibitors showed a relationship between increased expression of the LOX family and a reduced response to PD‐1/PD‐L1 treatment, which in turn led to worse overall survival results. Additionally, prior studies have shown that LOXL4 stimulated PD‐L1 activation, which resulted in the immunosuppressive phenotype of macrophages and the establishment of an immunosuppressive milieu that facilitated hepatocarcinogenesis [55]. All of these findings and confirmations pointed to the effectiveness of immunotherapy and chemotherapy in the treatment of malignancies.

## 5 Conclusions

This study suggested that the LOX family played a critical role in tumorigenesis and drug resistance, and that it was a risk factor for many types of cancer. Within the tumour microenvironment of many cancer types, there was a close relationship between various immune cell populations and elevated levels of LOX family members. As such, the LOX family had shown promise as a biomarker for predicting drug response. Our thorough investigation brought to light the possible therapeutic importance of LOX family‐specifically, LOX and LOXL2 in the advancement of cancer therapy strategies. Future studies should focus on the functional heterogeneity, synthetic lethal interactions and immunoregulatory mechanisms of LOX members and develop tissue microenvironment‐specific delivery systems to improve therapeutic precision. In addition, the combination of single‐cell sequencing and spatial transcriptome technologies is expected to reveal the dynamic regulatory maps of LOX in TME cell subpopulations, which will provide a theoretical basis for the design of combination therapies.

## Author Contributions

**Hongjin Liu:** conceptualization (equal), data curation (lead), formal analysis (lead), investigation (lead), methodology (equal), visualization (lead), writing – original draft (lead). **Xiaojiao Sun:** data curation (supporting), writing – original draft (supporting). **Bingqi Dong:** data curation (supporting). **Jixin Zhang:** methodology (supporting). **Junling Zhang:** data curation (supporting), investigation (supporting), writing – review and editing (supporting). **Yanlun Gu:** data curation (supporting), investigation (supporting). **Lin Chen:** data curation (supporting), investigation (supporting). **Xiaocong Pang:** conceptualization (equal), funding acquisition (supporting), writing – review and editing (equal). **Jingming Ye:** conceptualization (equal), data curation (equal), funding acquisition (supporting), writing – review and editing (equal). **Xin Wang:** conceptualization (equal), funding acquisition (equal), project administration (equal), writing – review and editing (equal). **Zhuona Rong:** conceptualization (lead), data curation (equal), funding acquisition (equal), methodology (lead), project administration (equal), validation (equal), writing – original draft (equal), writing – review and editing (lead).

## Ethics Statement

This study was approved by the Ethics Committee on Human Research of Peking University First Hospital under authorisation number 2018–15.

## Consent

The authors have nothing to report.

## Conflicts of Interest

The authors declare no conflicts of interest.

## Supporting information


Figures S1–S4.

Tables S1–S9.

## Data Availability

All data and associated protocols are included in the Manuscript and Supporting Information and available to the readers.
