# Supplementary material for: Systematic Characterisation and Analysis of Lysyl Oxidase Family Members as Drivers of Tumour Progression and Multiple Drug Resistance
Source: J Cell Mol Med. 2025 Apr 3;29(7):e70536. doi: 10.1111/jcmm.70536 (PMC11967703; doi:10.1111/jcmm.70536)
Supplement: Supplementary file 1 — Figures S1–S4. [file JCMM-29-e70536-s001.pdf]

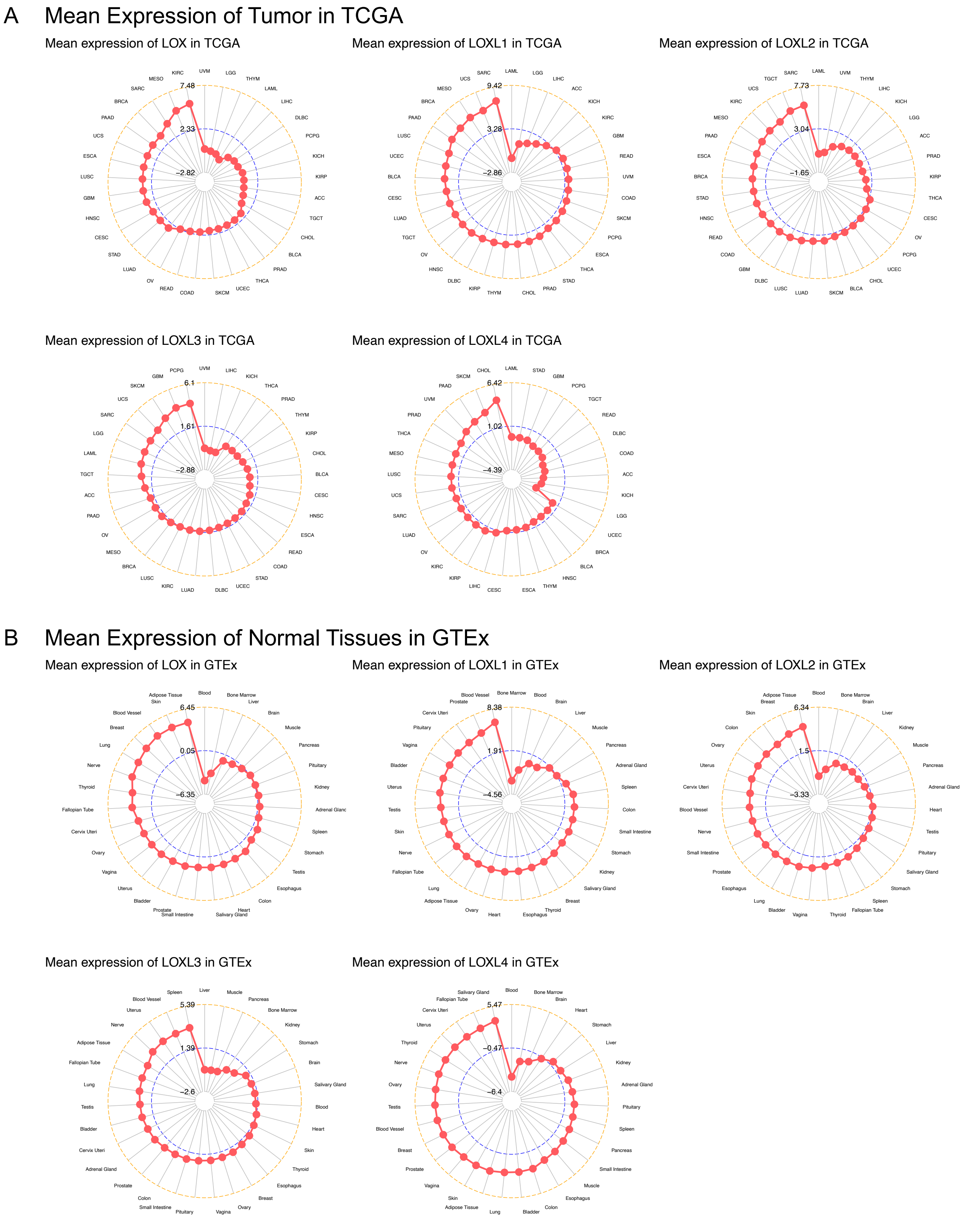

Figure S1. The expression of LOX family. *p < 0.05; **p < 0.01; ***p < 0.001 and ****p < 0.0001; ns, non-significant.

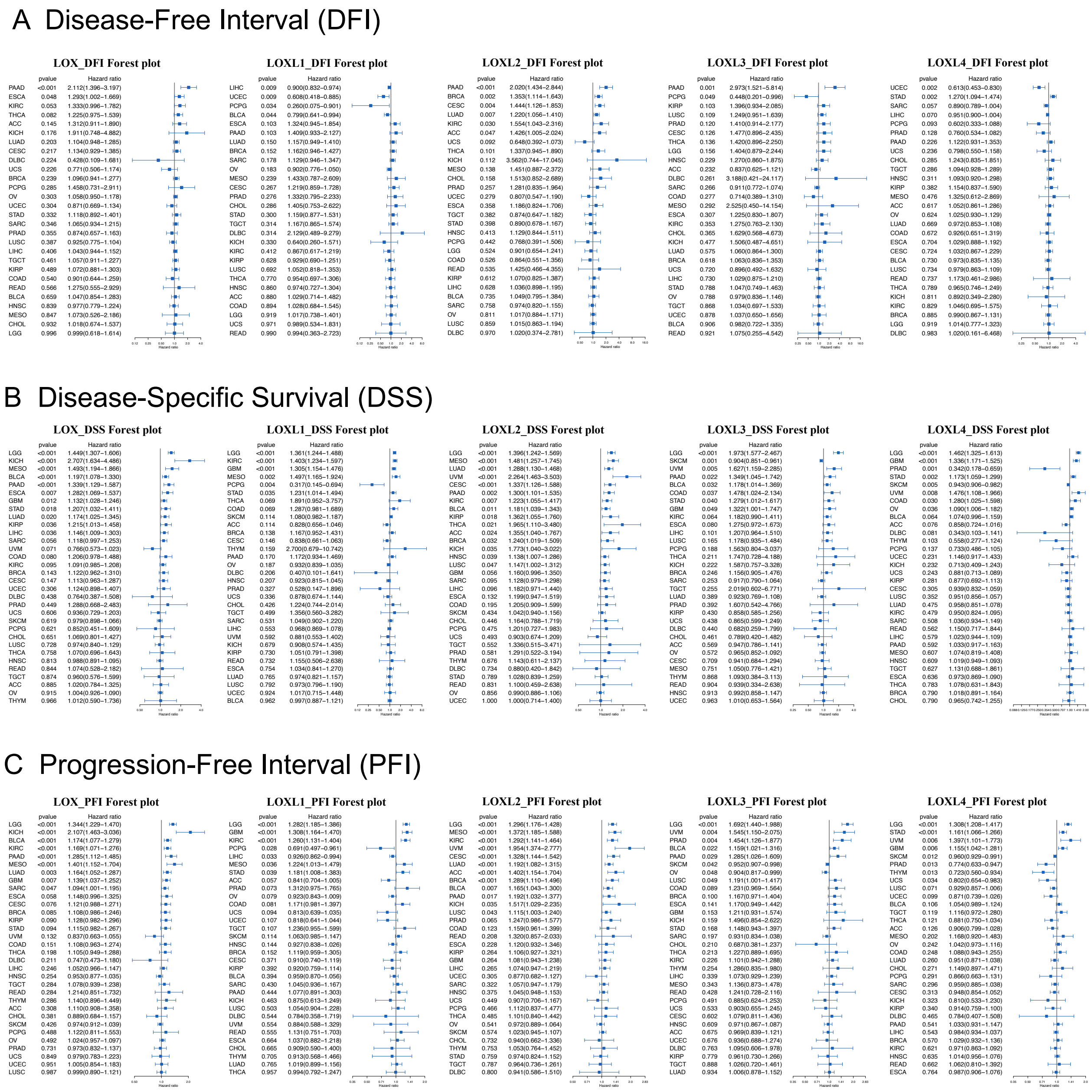

Figure S2. The uniCox analysis of LOX family.

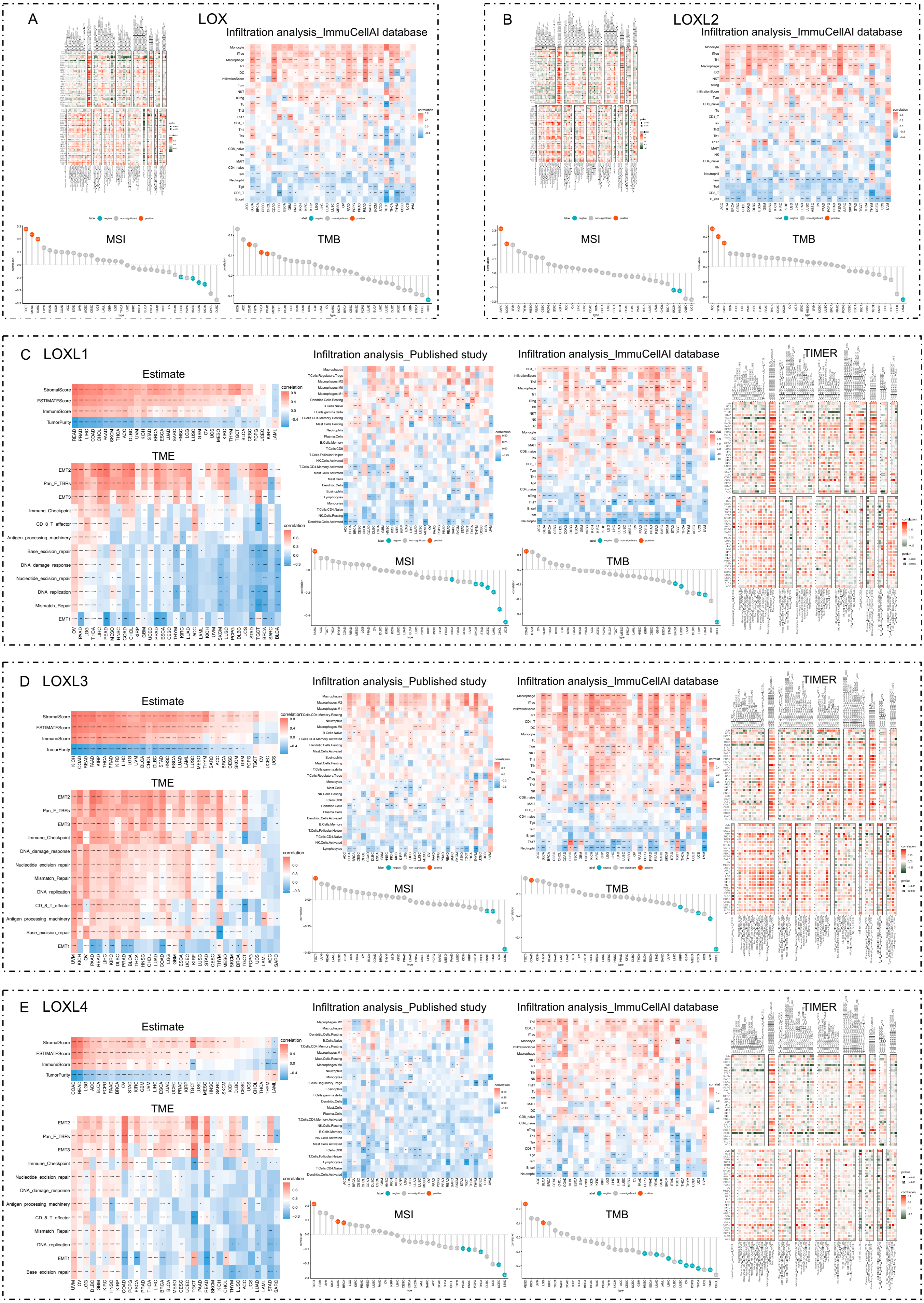

Figure S3. The correlation between LOX family and Tumor Microenvironment (TME), Immune Cell Infiltration, Tumor Mutation Burden (TMB) and Microsatellite Instability (MSI) . *$p < 0.05$; **$p < 0.01$; ***$p < 0.001$ and ****$p < 0.0001$; ns, non-significant.

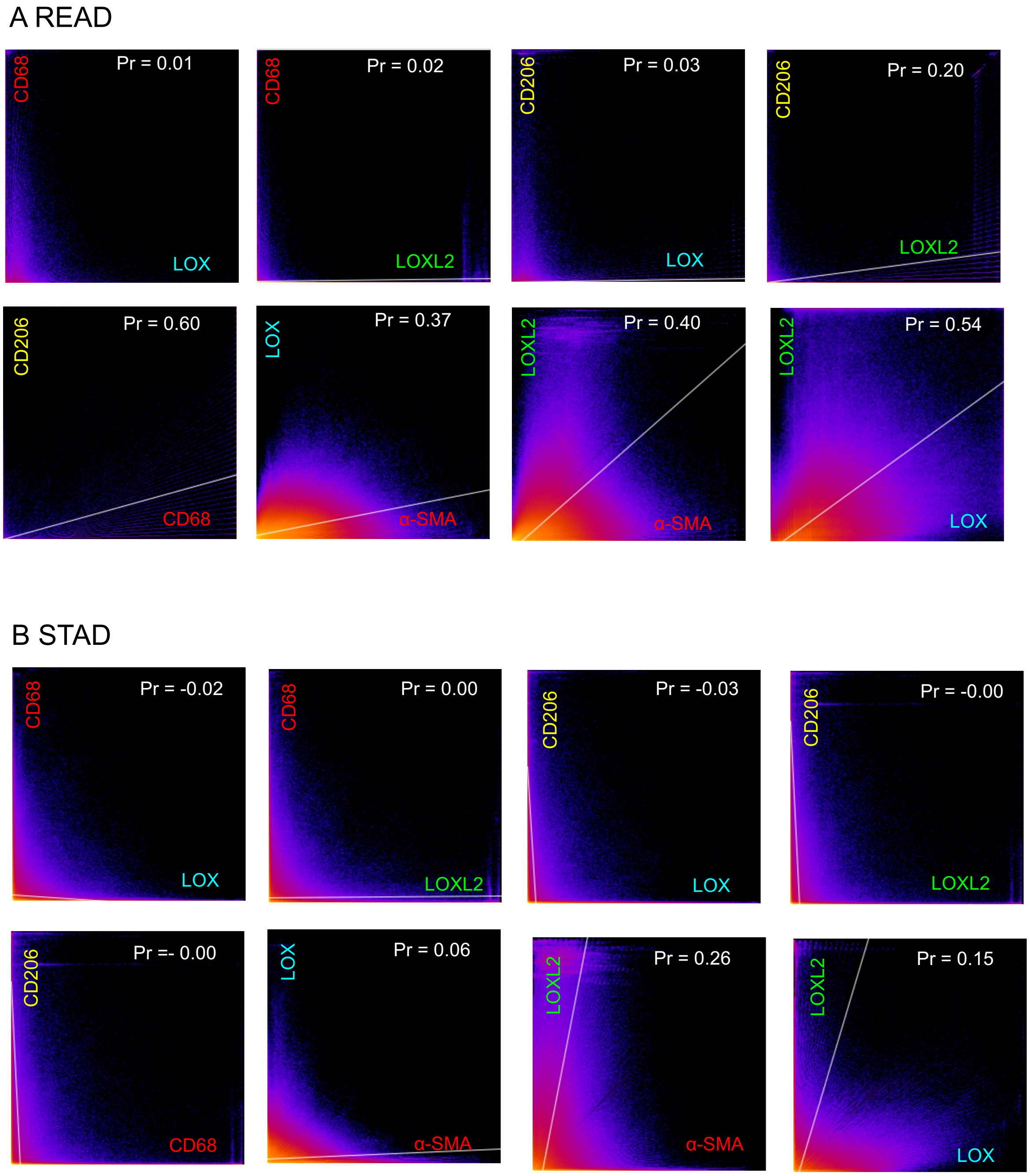

Figure S4. The immunofluorescence colocalizatio of READ and STAD.

Figure 4. The relationship between the LOX family and tumor microenvironment (TME) and immune infiltration
