# Supplementary material for: Systematic Characterisation and Analysis of Lysyl Oxidase Family Members as Drivers of Tumour Progression and Multiple Drug Resistance
Source: J Cell Mol Med. 2025 Apr 3;29(7):e70536. doi: 10.1111/jcmm.70536 (PMC11967703; doi:10.1111/jcmm.70536)
Supplement: Supplementary file 2 — Tables S1–S9. [file JCMM-29-e70536-s002.pdf]

Table S1 **Specific details regarding medication and clinical data for each patient and organoid**

Tumor Type	Preoperative therapy	Cycle of treatment	Postoperative pathological grading
BRCA (TNBC)	TA	6	Miller-Payne Grade 1 (poor response)
BRCA (TNBC)	TA	6	Miller-Payne Grade 1 (poor response)
BRCA (TNBC)	TA	6	Miller-Payne Grade 1 (poor response)
BRCA (TNBC)	TA	5	Miller-Payne Grade 1 (poor response)
BRCA (TNBC)	TA	6	Miller-Payne Grade 1 (poor response)
BRCA (TNBC)	TA	6	Miller-Payne Grade 1 (poor response)
BRCA (TNBC)	TA	6	Miller-Payne Grade 1 (poor response)
BRCA (TNBC)	TA→NP	2→6	Miller-Payne Grade 1 (poor response)
BRCA (TNBC)	TA→NP	2→5	Miller-Payne Grade 1 (poor response)
BRCA (TNBC)	TA→NP	4→2	Miller-Payne Grade 1 (poor response)
BRCA (TNBC)	TA	6	Miller-Payne Grade 5 (complete response)
BRCA (TNBC)	TA	5	Miller-Payne Grade 5 (complete response)
BRCA (TNBC)	TA	6	Miller-Payne Grade 5 (complete response)
BRCA (TNBC)	TA	3	Miller-Payne Grade 5 (complete response)
BRCA (TNBC)	TA	6	Miller-Payne Grade 5 (complete response)
BRCA (TNBC)	TA	6	Miller-Payne Grade 5 (complete response)
BRCA (TNBC)	TA	6	Miller-Payne Grade 5 (complete response)
BRCA (TNBC)	TA	6	Miller-Payne Grade 5 (complete response)
BRCA (TNBC)	TA	6	Miller-Payne Grade 5 (complete response)
BRCA (TNBC)	TA→NP	4→2	Miller-Payne Grade 5 (complete response)
READ	Xelox→mFOLFOX6 + Cetuximab	6	CAP Grade 3 (poor response)
READ	Xelox + Bevacizumab	5	CAP Grade 0 (complete response)
STAD	SOX + PD-1 inhibitor	4	CAP Grade 3 (poor response)
STAD	Xelox + PD-1 inhibitor	6	CAP Grade 0 (complete response)

Organoid Tumor Type	CytoMap Number	Chemotherapy	Cycle of Treatment	Endocrinotherapy	Time of Treatment	Organodis Drug Sensitivity
Breast Cancer	KO-16554	TC	6	Toremifene	8 months	Fulvestrant (IC50=1) ; Palbociclib (IC50=0.006)
Breast Cancer	KO-49857	AC-T	8	Letrozole	6 months	Fulvestrant (IC50=2.568)
Breast Cancer	KO-80803	ND	ND	ND	ND	Fulvestrant (IC50=42.46)
Breast Cancer	KO-89700	ND	ND	ND	ND	Fulvestrant (IC50=585.9)

Table S2 Tumor types and their abbreviation

Abbreviation	Tumor Types
ACC	Adrenocortical Carcinoma
BLCA	Bladder Urothelial Carcinoma
BRCA	Breast Invasive Carcinoma
CESC	Cervical Squamous Cell Carcinoma and Endocervical Adenocarcinoma
CHOL	Cholangiocarcinoma
COAD	Colorectal Adenocarcinoma
DLBC	Diffuse Large B-Cell Lymphoma
ESCA	Esophageal Carcinoma
GBM	Glioblastoma Multiforme
HNSC	Head and Neck Squamous Cell Carcinoma
KICH	Kidney Chromophobe
KIRC	Kidney Renal Clear Cell Carcinoma
KIRP	Kidney Renal Papillary Cell Carcinoma
LAML	Acute Myeloid Leukemia
LGG	Brain Lower Grade Glioma
LIHC	Liver Hepatocellular Carcinoma
LUAD	Lung Adenocarcinoma
LUSC	Lung Squamous Cell Carcinoma
MESO	Mesothelioma
OV	Ovarian Serous Cystadenocarcinoma
PAAD	Pancreatic Adenocarcinoma
PCPG	Pheochromocytoma and Paraganglioma
PRAD	Prostate Adenocarcinoma
READ	Rectum Adenocarcinoma
SARC	Sarcoma
SKCM	Skin Cutaneous Melanoma
STAD	Stomach Adenocarcinoma
TGCT	Testicular Germ Cell Tumors
THCA	Thyroid Carcinoma
THYM	Thymoma
UCEC	Uterine Corpus Endometrial Carcinoma
UCS	Uterine Carcinosarcoma
UVM	Uveal Melanoma

Table S3 The comparison of LOX family expression between tumor tissue and normal tissue, as well as tumor tissue and paired adjacent t

Tumor Type	Tumor Tissue versus Normal Tissue					Tumor Tissue versus Paired Adjacent Tissue				
	LOX	LOXL1	LOXL2	LOXL3	LOXL4	LOX	LOXL1	LOXL2	LOXL3	LOXL4
ACC	ND	ND	ND	ND	ND	ND	ND	ND	ND	ND
BLCA	NS	NS	HIGH (*)	LOW (***)	LOW (*)	NS	NS	HIGH (*)	NS	NS
BRCA	LOW (**)	HIGH (****)	HIGH (****)	HIGH (****)	LOW (****)	NS	HIGH (****)	HIGH (**)	HIGH (**)	LOW (****)
CESC	NS	NS	NS	NS	LOW (***)	NS	NS	NS	NS	NS
CHOL	HIGH (****)	HIGH (****)	HIGH (****)	HIGH (****)	HIGH (****)	HIGH (***)	HIGH (****)	HIGH (***)	HIGH (***)	HIGH (**)
COAD	HIGH (****)	HIGH (***)	HIGH (****)	NS	LOW (****)	HIGH (***)	NS	HIGH (****)	NS	LOW (****)
DLBC	ND	ND	ND	ND	ND	ND	ND	ND	ND	ND
ESCA	HIGH (**)	HIGH (*)	HIGH (***)	HIGH (*)	NS	NS	HIGH (*)	HIGH (**)	HIGH (*)	NS
GBM	ND	ND	ND	ND	ND	ND	ND	ND	ND	ND
HNSC	HIGH (****)	HIGH (****)	HIGH (****)	HIGH (****)	NS	HIGH (****)	HIGH (***)	HIGH (****)	HIGH (****)	NS
KICH	LOW (*)	LOW (****)	NS	LOW (****)	LOW (****)	NS	LOW (***)	NS	LOW (****)	LOW (****)
KIRC	HIGH (****)	LOW (****)	HIGH (****)	HIGH (****)	LOW (****)	HIGH (****)	****	HIGH (****)	HIGH (****)	LOW (***)
KIRP	LOW (***)	NS	HIGH (**)	HIGH (*)	LOW (***)	NS	NS	HIGH (*)	NS	LOW (*)
LAML	ND	ND	ND	ND	ND	ND	ND	ND	ND	ND
LGG	ND	ND	ND	ND	ND	ND	ND	ND	ND	ND
LIHC	HIGH (****)	HIGH (****)	HIGH (****)	HIGH (**)	HIGH (***)	HIGH (****)	HIGH (*)	HIGH (****)	NS	NS
LUAD	NS	HIGH (****)	HIGH (****)	NS	LOW (*)	HIGH (***)	HIGH (****)	HIGH (****)	NS	NS
LUSC	NS	HIGH (****)	HIGH (****)	NS	NS	HIGH (**)	HIGH (****)	HIGH (****)	NS	NS
MESO	ND	ND	ND	ND	ND	ND	ND	ND	ND	ND
OV	ND	ND	ND	ND	ND	ND	ND	ND	ND	ND
PAAD	NS	NS	NS	NS	NS	NS	NS	NS	NS	NS
PCPG	NS	HIGH (**)	HIGH (*)	HIGH (*)	LOW (*)	NS	NS	HIGH (**)	NS	NS
PRAD	HIGH (*)	LOW (****)	LOW (****)	LOW (**)	LOW (**)	HIGH (**)	****	LOW (*)	LOW (*)	NS
READ	HIGH (*)	NS	HIGH (***)	NS	LOW (**)	NS	NS	NS	NS	NS
SARC	HIGH (*)	NS	NS	NS	NS	NS	NS	NS	HIGH (*)	NS
SKCM	NS	ND	ND	ND	ND	ND	ND	ND	ND	ND
STAD	HIGH (****)	HIGH (****)	HIGH (****)	HIGH (****)	NS	HIGH (****)	HIGH (****)	HIGH (****)	HIGH (*)	NS
TGCT	ND	ND	ND	ND	ND	ND	ND	ND	ND	ND
THCA	NS	HIGH (****)	HIGH (****)	NS	LOW (****)	NS	HIGH (**)	HIGH (****)	NS	LOW (**)
THYM	NS	NS	NS	NS	NS	NS	NS	NS	NS	NS
UCEC	NS	HIGH (*)	NS	NS	LOW (****)	NS	HIGH (*)	NS	NS	LOW (*)
UCS	ND	ND	ND	ND	ND	ND	ND	ND	ND	ND
UVM	ND	ND	ND	ND	ND	ND	ND	ND	ND	ND

NS, non-significant; ND, no data. *$p < 0.05$; **$p < 0.01$; ***$p < 0.001$ and ****$p < 0.0001$.

Table S4 Correlation between the expression of LOX family and clinical tumor stages.

Tumor Type	I+II versus III+IV					I, II, III, IV compare with each other				
	LOX	LOXL1	LOXL2	LOXL3	LOXL4	LOX	LOXL1	LOXL2	LOXL3	LOXL4
ACC	**	NS	NS	NS	NS	*	NS	NS	*	NS
BLCA	****	NS	****	****	*	****	NS	****	****	NS
BRCA	NS	*	NS	NS	NS	NS	**	NS	NS	NS
CESC	NS	*	NS	NS	*	NS	**	NS	NS	**
CHOL	NS	*	NS	NS	NS	NS	NS	NS	NS	NS
COAD	NS	NS	*	NS	*	*	NS	*	*	*
DLBC	NS	NS	NS	NS	NS	NS	NS	*	NS	NS
ESCA	NS	NS	NS	NS	NS	**	NS	NS	*	**
GBM	ND	ND	ND	ND	ND	ND	ND	ND	ND	ND
HNSC	NS	NS	NS	NS	NS	**	NS	NS	NS	NS
KICH	*	NS	NS	NS	NS	*	NS	NS	NS	NS
KIRC	**	***	NS	NS	NS	*	**	NS	NS	*
KIRP	*	NS	*	NS	NS	*	NS	*	NS	NS
LAML	ND	ND	ND	ND	ND	ND	ND	ND	ND	ND
LGG	ND	ND	ND	ND	ND	ND	ND	ND	ND	ND
LIHC	**	NS	*	NS	NS	***	NS	*	*	NS
LUAD	*	NS	**	NS	NS	**	*	***	NS	*
LUSC	NS	NS	NS	NS	*	NS	NS	NS	NS	*
MESO	NS	NS	NS	*	NS	**	NS	NS	*	NS
OV	NS	NS	NS	*	NS	NS	NS	NS	**	NS
PAAD	NS	NS	NS	NS	NS	NS	NS	NS	NS	NS
PCPG	ND	ND	ND	ND	ND	ND	ND	ND	ND	ND
PRAD	ND	ND	ND	ND	ND	ND	ND	ND	ND	ND
READ	NS	NS	NS	NS	NS	NS	NS	*	*	*
SARC	ND	ND	ND	ND	ND	ND	ND	ND	ND	ND
SKCM	NS	NS	NS	NS	NS	NS	NS	NS	NS	*
STAD	NS	*	NS	NS	NS	**	***	NS	NS	*
TGCT	NS	NS	NS	NS	NS	NS	NS	NS	NS	NS
THCA	****	****	0	NS	NS	****	****	NS	*	NS
THYM	ND	ND	ND	ND	ND	ND	ND	ND	ND	ND
UCEC	NS	NS	NS	NS	NS	NS	NS	NS	NS	NS
UCS	*	NS	NS	NS	NS	*	NS	NS	*	*
UVM	NS	NS	**	NS	*	NS	NS	**	NS	***

NS, non-significant; ND, no data. *$p < 0.05$; **$p < 0.01$; ***$p < 0.001$ and ****$p < 0.0001$.

Table S5 The uniCox analysis of LOX family

Tumor Type	OS_COX forest					DFI_COX forest					DSS_COX forest					PFI_COX forest				
	LOX	LOXL1	LOXL2	LOXL3	LOXL4	LOX	LOXL1	LOXL2	LOXL3	LOXL4	LOX	LOXL1	LOXL2	LOXL3	LOXL4	LOX	LOXL1	LOXL2	LOXL3	LOXL4
ACC			+					+					+					+		
BLCA	+		+	+			-				+		+	+		+		+	+	
BRCA				+				+					+					+		
CESC			+					+					+					+		
CHOL																				
COAD														+	+					
DLBC																				
ESCA						+					+									
GBM	+	+		+	+						+	+		+	+	+	+			+
HNSC													+							
KICH	+										+		+			+		+		
KIRC		+		+				+				+	+			+	+	+		
KIRP	+		+								+		+							
LAML					-															
LGG	+	+	+	+	+						+	+	+	+	+	+	+	+	+	+
LIHC	+			+			-				+						-			
LUAD			+					+			+		+			+		+		
LUSC													+					+	+	
MESO	+	+	+								+	+	+			+	+	+		
OV					+										+				-	
PAAD	+		+			+		+	+		+		+	+		+		+	+	
PCPG		-					-		-			-					-			
PRAD					-										-				+	-
READ																				
SARC	+		+													+				
SKCM				-	-									-	-				-	-
STAD	+	+		+	+					+	+	+		+	+		+			+
TGCT																				
THCA		+	+										+							
THYM																				-
UCEC							-			-										
UCS																				-
UVM	-		+	+	+								+	+	+			+	+	+

+, positive correlation; -, negative correlation

Table S6 The Kaplan-Meier analysis of LOX family

Tumor Type	OS (Overall Survival)_Median					OS (Overall Survival)_optimal cut-off				
	LOX	LOXL1	LOXL2	LOXL3	LOXL4	LOX	LOXL1	LOXL2	LOXL3	LOXL4
ACC			+				-	+		-
BLCA	+					+		+	+	+
BRCA									+	
CESC			+			+	-	+	-	
CHOL							+		-	
COAD						+	+	+	+	+
DLBC							-		-	
ESCA		-				+	-		+	-
GBM	+				+	+	+	+	+	+
HNSC								+		+
KICH	+					+				-
KIRC		+				+	+	+	+	
KIRP	+		+			+	+	+		+
LAML					-				-	-
LGG	+	+	+	+	+	+	+	+	+	+
LIHC	+			+	+	+		+	+	+
LUAD			+			+	+	+	-	
LUSC			+			+		+		
MESO	+	+	+			+	+	+	+	+
OV					+	-		-		+
PAAD	+		+			+		+	+	
PCPG							-	+		-
PRAD										-
READ							+			+
SARC	+					+	+	+	-	+
SKCM				-	-		+		-	-
STAD	+				+	+	+	+	+	+
TGCT							+		+	
THCA						+	+	+	+	+
THYM				+		+	+		+	
UCEC								+	+	+
UCS							-			-
UVM	-		+		+	-		+	+	+

Tumor Type	DFI (Disease Free Interval)_Median					DFI (Disease Free Interval)_optimal cut-off				
	LOX	LOXL1	LOXL2	LOXL3	LOXL4	LOX	LOXL1	LOXL2	LOXL3	LOXL4
ACC								+	-	
BLCA							-	-		
BRCA			+			+		+		
CESC			+			+	+	+	+	
CHOL						+		+		
COAD								-	-	
DLBC						-				-
ESCA						+	+		+	
GBM										
HNSC									+	
KICH						+	-	+		
KIRC						+		+		
KIRP				+		+			+	+
LAML										
LGG							+		+	
LIHC							-			-
LUAD							+	+		
LUSC									+	
MESO							+	+	+	
OV						+				
PAAD	+		+	+		+	+	+	+	+
PCPG							-		+	-
PRAD							+	+	+	
READ										
SARC						+	+	-	-	-
SKCM										
STAD					+					+
TGCT						+	+			
THCA	+					+	-	+		
THYM										
UCEC					-		-			-
UCS			-					-		
UVM										

Tumor Type	DSS (Disease Specific Survival)_Median					DSS (Disease Specific Survival)_optimal cut-off				
	LOX	LOXL1	LOXL2	LOXL3	LOXL4	LOX	LOXL1	LOXL2	LOXL3	LOXL4
ACC			+				-	+		-
BLCA	+					+		+	+	+
BRCA						+	+	+	+	
CESC			+			+	-	+	-	-
CHOL							+	+		
COAD				+	+	+	+	+	+	+
DLBC					-				-	-
ESCA	+		+			+		+	+	-
GBM	+	+			+	+	+	+	+	+
HNSC			+					+		+
KICH	+					+		+		-
KIRC		+	+			+	+	+		-
KIRP	+		+			+	+	+		-
LAML										
LGG	+	+	+	+	+	+	+	+	+	+
LIHC				+		+		+	+	
LUAD			+			+		+	-	
LUSC			+					+		
MESO	+	+	+			+	+	+		
OV					+			+		+
PAAD	+		+			+	+	+	+	
PCPG							-			-
PRAD									+	+
READ										
SARC	+					+	+	+	-	
SKCM				-	-		+		-	-
STAD	+				+	+	+			+
TGCT										
THCA							+	+	+	+
THYM							+			
UCEC						+			-	+
UCS							-			-
UVM			+	+	+	-		+	+	+

Tumor Type	PFI (Progression Free Interval)_Median					PFI (Progression Free Interval)_optimal cut-off				
	LOX	LOXL1	LOXL2	LOXL3	LOXL4	LOX	LOXL1	LOXL2	LOXL3	LOXL4
ACC			+			+	-	+		-
BLCA	+				+	+		+	+	+
BRCA			+			+		+	+	
CESC			+			+	-	+		
CHOL										
COAD		+			+	+	+	+	+	+
DLBC						-	+		-	-
ESCA			+			+		+	+	-
GBM		+			+	+	+	+		+
HNSC							-		-	
KICH	+		+			+		+		-
KIRC	+	+	+			+	-	+		-
KIRP						+	+			-
LAML										
LGG	+	+	+	+	+	+	+	+	+	+
LIHC						+	-	+	+	-
LUAD	+					+		+	-	
LUSC			+	+	-			+	+	-
MESO	+	+	+			+	+	+	+	
OV						+	-	-	-	+
PAAD	+					+	+	+	+	
PCPG							-			
PRAD				+			+	+	+	+
READ								+		
SARC						+	+	+	-	-
SKCM				-	-	+	+		-	-
STAD	+				+	+	+			+
TGCT						+	+	+	+	+
THCA						+	-	+		-
THYM										-
UCEC							-	-	-	-
UCS							-	-		-
UVM			+	+		-		+	+	+

+, positive correlation; -, negative correlation

Table S7 The Kaplan-Meier analysis of LOX family mrthylation

Tumor Type	OS_Methylation					DFI_Methylation					DSS_Methylation					PFI_Methylation				
	LOX	LOXL1	LOXL2	LOXL3	LOXL4	LOX	LOXL1	LOXL2	LOXL3	LOXL4	LOX	LOXL1	LOXL2	LOXL3	LOXL4	LOX	LOXL1	LOXL2	LOXL3	LOXL4
ACC		+		+	+															
BLCA	-							+												
BRCA									-					-					-	
CESC																				
CHOL														+			+			
COAD																				
DLBC																				
ESCA																				-
GBM			-																	
HNSC										+			-				+	-		
KICH																			-	
KIRC			-					-												
KIRP				-										-					-	
LAML				+																
LGG	-	-	-	-	-						-	-	-	-		-	-		-	-
LIHC							+													
LUAD															+					
LUSC											+									
MESO	-	-	-					-					-					-		
OV																				
PAAD		-	-								-					-				
PCPG																				
PRAD											-									
READ																				
SARC															+					
SKCM				+																
STAD			-		-							-		-			-			
TGCT																-				
THCA				-		-										-				
THYM	-	-		-														+		
UCEC																				
UCS									-											
UVM		+	-										-							

+, positive correlation; -, negative correlation

Table S8 The relationship between the LOX family and the IC50 values of 192 antitumor drugs

Antitumor Drug	Correlation				
	LOX	LOXL1	LOXL2	LOXL3	LOXL4
**5-Fluorouracil**	positive	positive	positive	non-significant	positive
**ABT737**	positive	positive	positive	non-significant	positive
**Acetalax**	positive	positive	positive	positive	positive
**Afatinib**	positive	positive	positive	positive	non-significant
**Afuresertib**	positive	positive	positive	non-significant	positive
**AGI-5198**	positive	positive	positive	non-significant	positive
**AGI-6780**	positive	positive	positive	non-significant	positive
**Alisertib**	positive	positive	positive	non-significant	non-significant
**Alpelisib**	positive	positive	positive	non-significant	non-significant
**AMG-319**	positive	positive	non-significant	non-significant	positive
**AT13148**	non-significant	non-significant	non-significant	non-significant	positive
**Axitinib**	non-significant	non-significant	non-significant	non-significant	non-significant
**AZ6102**	positive	positive	positive	non-significant	positive
**AZ960**	non-significant	non-significant	non-significant	non-significant	non-significant
**AZD1208**	positive	positive	positive	non-significant	positive
**AZD1332**	negtive	non-significant	negtive	non-significant	non-significant
**AZD2014**	non-significant	non-significant	non-significant	non-significant	non-significant
**AZD3759**	positive	positive	positive	positive	non-significant
**AZD4547**	non-significant	non-significant	positive	non-significant	positive
**AZD5153**	positive	positive	positive	non-significant	non-significant
**AZD5363**	non-significant	positive	non-significant	non-significant	positive
**AZD5438**	positive	positive	positive	non-significant	non-significant
**AZD5582**	positive	positive	positive	non-significant	non-significant
**AZD5991**	positive	positive	positive	non-significant	positive
**AZD6482**	non-significant	non-significant	non-significant	non-significant	non-significant
**AZD6738**	positive	positive	positive	non-significant	positive
**AZD7762**	positive	positive	positive	non-significant	positive
**AZD8055**	non-significant	non-significant	non-significant	non-significant	non-significant
**AZD8186**	non-significant	non-significant	non-significant	non-significant	non-significant
**BDP-00009066**	positive	positive	non-significant	non-significant	positive
**BI-2536**	non-significant	non-significant	non-significant	non-significant	non-significant
**BIBR-1532**	positive	positive	positive	non-significant	positive
**BMS-345541**	positive	positive	positive	non-significant	positive
**BMS-536924**	non-significant	non-significant	non-significant	non-significant	non-significant
**BMS-754807**	non-significant	non-significant	non-significant	positive	non-significant
**Bortezomib**	non-significant	positive	non-significant	negtive	positive
**BPD-00008900**	non-significant	positive	non-significant	non-significant	positive
**Buparlisib**	positive	positive	positive	non-significant	positive
**Camptothecin**	positive	positive	positive	non-significant	positive
**Carmustine**	positive	positive	positive	non-significant	positive
**CDK9_5038**	positive	positive	positive	non-significant	non-significant
**CDK9_5576**	positive	positive	positive	non-significant	non-significant
**Cediranib**	non-significant	non-significant	non-significant	non-significant	positive
**Cisplatin**	positive	positive	positive	non-significant	positive
**Crizotinib**	positive	positive	positive	non-significant	positive
**Cyclophosphamide**	positive	positive	positive	non-significant	positive
**Cytarabine**	positive	positive	positive	non-significant	positive
**CZC24832**	positive	positive	positive	non-significant	positive
**Dabrafenib**	positive	positive	positive	non-significant	positive
**Dactinomycin**	positive	positive	positive	non-significant	positive
**Dactolisib**	non-significant	positive	non-significant	non-significant	non-significant
**Daporinad**	positive	non-significant	positive	non-significant	non-significant

**Dasatinib**	negtive	non-significant	negtive	positive	non-significant
**Dihydrorotenone**	positive	positive	positive	non-significant	non-significant
**Dinaciclib**	positive	positive	positive	non-significant	positive
**Docetaxel**	positive	positive	positive	non-significant	positive
**Doramapimod**	non-significant	non-significant	non-significant	non-significant	non-significant
**Eg5_9814**	positive	positive	positive	non-significant	positive
**Elephantin**	positive	positive	positive	non-significant	non-significant
**Entinostat**	positive	positive	positive	negtive	positive
**Entospletinib**	non-significant	positive	negtive	non-significant	non-significant
**Epirubicin**	positive	positive	non-significant	non-significant	positive
**EPZ004777**	positive	positive	positive	non-significant	positive
**EPZ5676**	positive	positive	positive	non-significant	positive
**ERK_2440**	non-significant	non-significant	non-significant	negtive	non-significant
**ERK_6604**	positive	positive	positive	non-significant	non-significant
**Erlotinib**	positive	positive	positive	positive	non-significant
**Fludarabine**	positive	positive	positive	non-significant	positive
**Foretinib**	non-significant	non-significant	non-significant	negtive	positive
**Fulvestrant**	positive	positive	positive	non-significant	positive
**Gallibiscoquinazole**	positive	positive	positive	non-significant	positive
**GDC0810**	positive	positive	positive	non-significant	positive
**Gefitinib**	positive	positive	positive	positive	non-significant
**Gemcitabine**	positive	positive	positive	non-significant	non-significant
**GNE-317**	non-significant	non-significant	non-significant	non-significant	non-significant
**GSK1904529A**	positive	positive	positive	non-significant	positive
**GSK2578215A**	positive	positive	positive	non-significant	positive
**GSK2606414**	positive	positive	positive	non-significant	positive
**GSK269962A**	non-significant	non-significant	non-significant	non-significant	non-significant
**GSK343**	positive	positive	positive	non-significant	positive
**GSK591**	positive	positive	positive	non-significant	positive
**IAP_5620**	positive	positive	positive	non-significant	positive
**I-BET-762**	positive	positive	positive	non-significant	non-significant
**I-BRD9**	positive	positive	positive	non-significant	positive
**Ibrutinib**	positive	positive	positive	positive	non-significant
**IGF1R_3801**	non-significant	non-significant	non-significant	non-significant	non-significant
**Ipatasertib**	positive	positive	positive	non-significant	positive
**IRAK4_4710**	positive	positive	positive	non-significant	positive
**Irinotecan**	positive	positive	positive	negtive	positive
**IWP-2**	positive	positive	positive	non-significant	positive
**JAK_8517**	non-significant	non-significant	non-significant	non-significant	non-significant
**JAK1_8709**	positive	positive	positive	non-significant	positive
**JQ1**	non-significant	non-significant	non-significant	non-significant	non-significant
**KRAS (G12C) Inhibitor-12**	positive	positive	positive	non-significant	positive
**KU-55933**	non-significant	non-significant	non-significant	non-significant	non-significant
**Lapatinib**	positive	positive	positive	positive	positive
**LCL161**	positive	positive	positive	non-significant	non-significant
**Leflunomide**	positive	positive	positive	non-significant	positive
**LGK974**	positive	positive	positive	non-significant	positive
**Linsitinib**	positive	positive	positive	non-significant	non-significant
**LJI308**	positive	positive	positive	non-significant	positive
**Luminespib**	non-significant	positive	negtive	non-significant	non-significant
**LY2109761**	positive	positive	positive	non-significant	positive
**MG-132**	non-significant	positive	non-significant	negtive	positive
**MIM1**	positive	positive	positive	non-significant	positive
**MIRA-1**	positive	positive	positive	non-significant	positive
**Mirin**	positive	positive	positive	non-significant	non-significant
**Mitoxantrone**	positive	positive	positive	non-significant	non-significant

**MK-1775**	positive	positive	positive	non-significant	positive
**MK-2206**	positive	positive	positive	non-significant	positive
**MK-8776**	positive	positive	positive	non-significant	positive
**ML323**	positive	positive	positive	non-significant	positive
**MN-64**	positive	positive	positive	non-significant	positive
**Navitoclax**	positive	positive	positive	non-significant	positive
**Nelarabine**	positive	positive	positive	non-significant	positive
**Nilotinib**	positive	positive	positive	non-significant	positive
**Niraparib**	positive	positive	positive	negtive	positive
**NU7441**	non-significant	non-significant	non-significant	non-significant	non-significant
**Nutlin-3a (-)**	positive	positive	non-significant	negtive	positive
**NVP-ADW742**	positive	positive	positive	negtive	positive
**Obatoclax Mesylate**	positive	positive	non-significant	non-significant	non-significant
**OF-1**	positive	positive	positive	non-significant	positive
**Olaparib**	positive	non-significant	positive	non-significant	positive
**OSI-027**	positive	non-significant	non-significant	non-significant	positive
**Osimertinib**	positive	positive	positive	positive	non-significant
**OTX015**	positive	positive	non-significant	non-significant	non-significant
**Oxaliplatin**	positive	positive	positive	non-significant	positive
**P22077**	positive	positive	positive	non-significant	positive
**Paclitaxel**	positive	positive	positive	non-significant	positive
**PAK_5339**	positive	positive	positive	non-significant	positive
**Palbociclib**	positive	positive	positive	non-significant	non-significant
**PCI-34051**	positive	positive	positive	negtive	positive
**PD0325901**	positive	positive	positive	non-significant	negtive
**PD173074**	positive	positive	positive	non-significant	positive
**Pevonedistat**	positive	positive	positive	non-significant	positive
**PF-4708671**	non-significant	non-significant	non-significant	non-significant	non-significant
**PFI3**	positive	positive	positive	non-significant	positive
**Picolinici-acid**	positive	positive	positive	non-significant	positive
**Pictilisib**	non-significant	positive	non-significant	non-significant	positive
**PLX-4720**	non-significant	positive	non-significant	negtive	non-significant
**Podophyllotoxin bromide**	positive	positive	positive	non-significant	positive
**PRIMA-1MET**	positive	positive	positive	non-significant	non-significant
**PRT062607**	positive	positive	positive	non-significant	non-significant
**Pyridostatin**	positive	positive	non-significant	non-significant	positive
**Rapamycin**	positive	positive	non-significant	non-significant	positive
**Ribociclib**	non-significant	non-significant	positive	non-significant	non-significant
**RO-3306**	non-significant	non-significant	non-significant	non-significant	non-significant
**Ruxolitinib**	positive	positive	positive	non-significant	positive
**RVX-208**	positive	positive	positive	non-significant	non-significant
**Sabutoclax**	positive	positive	positive	non-significant	positive
**Sapitinib**	positive	positive	positive	positive	non-significant
**Savolitinib**	positive	positive	positive	non-significant	positive
**SB216763**	non-significant	non-significant	non-significant	non-significant	non-significant
**SB505124**	negtive	non-significant	non-significant	positive	non-significant
**SCH772984**	positive	positive	positive	non-significant	negtive
**Selumetinib**	positive	positive	positive	non-significant	negtive
**Sepantronium bromide**	non-significant	non-significant	negtive	non-significant	non-significant
**Sinularin**	positive	positive	positive	non-significant	positive
**Sorafenib**	positive	positive	positive	non-significant	positive
**Staurosporine**	negtive	negtive	negtive	non-significant	non-significant
**TAF1_5496**	positive	positive	positive	non-significant	positive
**Talazoparib**	non-significant	positive	positive	negtive	positive
**Tamoxifen**	positive	positive	positive	non-significant	positive
**Taselisib**	positive	positive	positive	non-significant	non-significant

**Telomerase Inhibitor IX**	positive	positive	non-significant	negtive	non-significant
**Temozolomide**	positive	positive	positive	non-significant	positive
**Teniposide**	positive	positive	positive	non-significant	non-significant
**Topotecan**	positive	positive	positive	non-significant	non-significant
**Tozasertib**	negtive	non-significant	non-significant	non-significant	non-significant
**Trametinib**	non-significant	positive	non-significant	non-significant	negtive
**Ulixertinib**	positive	positive	positive	non-significant	non-significant
**ULK1_4989**	non-significant	non-significant	non-significant	non-significant	positive
**UMI-77**	positive	positive	positive	negtive	non-significant
**Uprosertib**	positive	positive	positive	non-significant	positive
**VE821**	positive	positive	positive	non-significant	positive
**VE-822**	positive	positive	positive	non-significant	positive
**Venetoclax**	positive	positive	positive	negtive	positive
**Vinblastine**	positive	positive	positive	non-significant	positive
**Vincristine**	positive	positive	positive	non-significant	positive
**Vinorelbine**	positive	positive	positive	non-significant	positive
**Vorinostat**	positive	positive	positive	negtive	positive
**VSP34_8731**	positive	positive	positive	non-significant	non-significant
**VX-11e**	positive	positive	positive	non-significant	non-significant
**Wee1 Inhibitor**	positive	positive	positive	non-significant	positive
**WEHI-539**	positive	non-significant	positive	non-significant	positive
**WIKI4**	non-significant	positive	non-significant	non-significant	non-significant
**Wnt-C59**	positive	positive	positive	non-significant	positive
**WZ4003**	positive	positive	non-significant	negtive	non-significant
**XAV939**	positive	positive	positive	non-significant	non-significant
**YK-4-279**	positive	positive	positive	non-significant	positive
**ZM447439**	non-significant	non-significant	non-significant	non-significant	non-significant
**Zoledronate**	positive	positive	positive	non-significant	positive

Table S9 The relationship between the LOX family and TMB or MSI

Tumor Type	MSI					TMB				
	LOX	LOXL1	LOXL2	LOXL3	LOXL4	LOX	LOXL1	LOXL2	LOXL3	LOXL4
ACC	non-significant	non-significant	non-significant	non-significant	non-significant	non-significant	non-significant	positive	negtive	non-significant
BLCA	non-significant	non-significant	non-significant	non-significant	non-significant	non-significant	non-significant	non-significant	non-significant	non-significant
BRCA	non-significant	negtive	non-significant	non-significant	positive	non-significant	non-significant	non-significant	non-significant	non-significant
CESC	non-significant	non-significant	non-significant	non-significant	non-significant	non-significant	non-significant	non-significant	non-significant	non-significant
CHOL	non-significant	negtive	non-significant	non-significant	non-significant	non-significant	negtive	non-significant	negtive	non-significant
COAD	non-significant	non-significant	non-significant	non-significant	non-significant	positive	positive	non-significant	positive	non-significant
DLBC	non-significant	non-significant	non-significant	non-significant	non-significant	non-significant	non-significant	non-significant	non-significant	non-significant
ESCA	non-significant	non-significant	non-significant	negtive	non-significant	non-significant	non-significant	non-significant	non-significant	non-significant
GBM	non-significant	non-significant	non-significant	non-significant	non-significant	non-significant	non-significant	non-significant	non-significant	non-significant
HNSC	non-significant	non-significant	non-significant	non-significant	non-significant	non-significant	non-significant	non-significant	non-significant	non-significant
KICH	negtive	non-significant	negtive	negtive	negtive	non-significant	non-significant	non-significant	non-significant	negtive
KIRC	non-significant	non-significant	non-significant	non-significant	non-significant	non-significant	non-significant	non-significant	non-significant	non-significant
KIRP	non-significant	non-significant	non-significant	non-significant	non-significant	non-significant	non-significant	non-significant	non-significant	non-significant
LAML	non-significant	non-significant	non-significant	non-significant	non-significant	negtive	non-significant	non-significant	negtive	non-significant
LGG	non-significant	negtive	non-significant	non-significant	non-significant	non-significant	non-significant	negtive	non-significant	non-significant
LIHC	non-significant	non-significant	non-significant	non-significant	non-significant	non-significant	non-significant	non-significant	non-significant	positive
LUAD	non-significant	non-significant	non-significant	non-significant	non-significant	non-significant	negtive	non-significant	non-significant	negtive
LUSC	negtive	non-significant	non-significant	non-significant	positive	non-significant	negtive	non-significant	non-significant	negtive
MESO	negtive	non-significant	non-significant	non-significant	non-significant	non-significant	negtive	non-significant	non-significant	negtive
OV	positive	non-significant	non-significant	non-significant	non-significant	non-significant	non-significant	non-significant	non-significant	positive
PAAD	non-significant	non-significant	non-significant	non-significant	non-significant	non-significant	non-significant	non-significant	non-significant	negtive
PCPG	non-significant	non-significant	non-significant	non-significant	non-significant	non-significant	non-significant	non-significant	non-significant	non-significant
PRAD	non-significant	non-significant	non-significant	non-significant	non-significant	non-significant	non-significant	non-significant	negtive	negtive
READ	non-significant	non-significant	non-significant	non-significant	negtive	positive	non-significant	non-significant	non-significant	non-significant
SARC	positive	positive	positive	non-significant	non-significant	non-significant	non-significant	positive	non-significant	non-significant
SKCM	negtive	negtive	negtive	non-significant	non-significant	non-significant	negtive	non-significant	non-significant	non-significant
STAD	non-significant	negtive	non-significant	negtive	negtive	non-significant	non-significant	non-significant	non-significant	negtive
TGCT	positive	non-significant	non-significant	positive	positive	non-significant	non-significant	non-significant	non-significant	non-significant
THCA	non-significant	non-significant	non-significant	non-significant	negtive	positive	non-significant	non-significant	non-significant	negtive
THYM	non-significant	non-significant	non-significant	non-significant	non-significant	non-significant	non-significant	positive	non-significant	non-significant
UCEC	non-significant	negtive	positive	non-significant	negtive	non-significant	non-significant	non-significant	non-significant	non-significant
UCS	non-significant	negtive	non-significant	non-significant	non-significant	non-significant	non-significant	non-significant	non-significant	non-significant
UVM	non-significant	non-significant	non-significant	non-significant	non-significant	non-significant	non-significant	non-significant	non-significant	non-significant
